# Functional Role of PPARs in Ruminants: Potential Targets for Fine-Tuning Metabolism during Growth and Lactation

**DOI:** 10.1155/2013/684159

**Published:** 2013-04-29

**Authors:** Massimo Bionaz, Shuowen Chen, Muhammad J. Khan, Juan J. Loor

**Affiliations:** ^1^Animal and Rangeland Sciences, Oregon State University, Corvallis, OR 97330, USA; ^2^Animal and Nutritional Sciences, University of Illinois, Urbana, IL 61801, USA

## Abstract

Characterization and biological roles of the peroxisome proliferator-activated receptor (PPAR) isotypes are well known in monogastrics, but not in ruminants. However, a wealth of information has accumulated in little more than a decade on ruminant PPARs including isotype tissue distribution, response to synthetic and natural agonists, gene targets, and factors affecting their expression. Functional characterization demonstrated that, as in monogastrics, the PPAR isotypes control expression of genes involved in lipid metabolism, anti-inflammatory response, development, and growth. Contrary to mouse, however, the PPAR*γ* gene network appears to controls milk fat synthesis in lactating ruminants. As in monogastrics, PPAR isotypes in ruminants are activated by long-chain fatty acids, therefore, making them ideal candidates for fine-tuning metabolism in this species via nutrients. In this regard, using information accumulated in ruminants and monogastrics, we propose a model of PPAR isotype-driven biological functions encompassing key tissues during the peripartal period in dairy cattle.

## 1. Introduction

In humans, mouse, and rat, nuclear receptors (NR), including PPARs, form a transcription factor family of 47–49 members [1]. Activity of NR allows for long-term (hours to days) control of metabolism because they can affect mRNA expression of target genes, including metabolic enzymes [2]. Thus, NR represent an important regulatory system in cells, tissues, and organs playing a central role in metabolic coordination of the entire organism.

Peroxisome proliferator-activated receptors (PPARs) were originally identified in *Xenopus* frogs [3] as novel members of the NR that induced the proliferation of peroxisomes in cells, a process that was accompanied by activation of the promoter of the acyl-CoA oxidase gene (*ACOX1*) encoding the key enzyme of peroxisomal long-chain fatty acid (LCFA) *β*-oxidation. The PPAR*α* was the first member or isotype of the PPARs to be discovered in mammals during the search of a molecular target for liver peroxisome proliferators [4]. Those compounds include hypolipidemic drugs, that is, fibrates (e.g., clofibrate, fenofibrate, or Wy-14643), whose main effect is to lower blood triacylglycerol (TAG) and regulate cholesterol concentrations [5].

Initial characterization of PPAR*α* (gene symbol *PPARA *in human and ruminants) in the adult mouse revealed that it was highly expressed in liver, kidney, and heart [4]. Shortly after PPAR*α* was discovered, the isotypes PPAR*γ* (gene symbol *PPARG*) and PPAR*β*/*δ* (gene symbol *PPARD*) were cloned [3, 6]. In monogastrics, *PPARA* is highly abundant in liver, intestine, heart, and kidney; *PPARG* is abundant in adipose and immune cells, while *PPARD* is ubiquitously expressed [7, 8]. In the mouse, both PPAR*γ* isoforms *γ*1 and *γ*2 act in white and brown adipose tissue to promote adipocyte differentiation and lipid storage. While PPAR*γ*2 is mainly expressed in adipocytes, PPAR*γ*1 is expressed at modest levels also in other cells/tissues [9]. Expression of PPAR*β*/*δ* in murine resembled closely that of PPAR*α* and was the sole isotype expressed in brain [6]. More recent studies in rats have established that PPAR*β*/*δ* is expressed ubiquitously throughout the body but is substantially more abundant in skeletal muscle than PPAR*α* or PPAR*γ* [7].

The PPARs form and function as heterodimers with retinoid-X-receptor (RXR). Once the ligand binds (e.g., LCFA, fibrates, thiazolidinedione (TZD)) to the ligand-binding domain (LBD), it produces a covalent modification of the PPAR structure [10] activating the NR. The activated PPAR/RXR binds to a specific DNA sequence (PPAR response element, PPRE) in the promoter region of specific target genes inducing or repressing their expression. The PPRE is a direct repeat of a hexanucleotide (AGGTCA) separated by a single nucleotide (i.e., DR-1). The DR-1 varies for each of the PPAR isotypes, thus conferring greater or lower strength to the PPAR/RXR complex for binding to PPRE and the strength of activation [11]. All PPAR isotypes are activated by ligand concentrations in the *μ*M range or below, at least in nonruminants [12–14].

## 2. Role of PPAR in Monogastrics

The PPAR isotypes play multiple roles in mammals. There are a vast number of excellent reviews discussing those aspects in detail (e.g., [2, 5, 15–19]). Among others, the PPAR isotypes play important roles in regulating lipid and glucose metabolism, controlling inflammatory response, regulating tissue repair and differentiation, and cancer progression. Although with contrasting roles, PPAR isotypes affect blood vessel formation [20]. The PPAR*γ* is pivotal in controlling the switch between adipogenesis and osteogenesis [17, 21] and insulin sensitivity [22], and it has an important neuroprotective role [23]. Similarly, it is well established that PPAR*α* plays a crucial role in hepatic fatty acid catabolism in mitochondria, peroxisome, and microsomes [18]. The PPAR*β*/*δ* controls fatty acid catabolism in skeletal muscle and heart [2]. The PPAR isotypes are known to play important roles in all the reproductive tissues studied to date (reviewed in [24]). Due to the important functions played by the PPAR isotypes, PPAR*α* and PPAR*γ* have long been considered promising drug targets for human metabolic disorders as they regulate lipid and/or glucose homeostasis by controlling uptake, synthesis, storage, and clearance [25].

## 3. PPAR Isotype Expression in Ruminant Tissues

**Figure 1:**
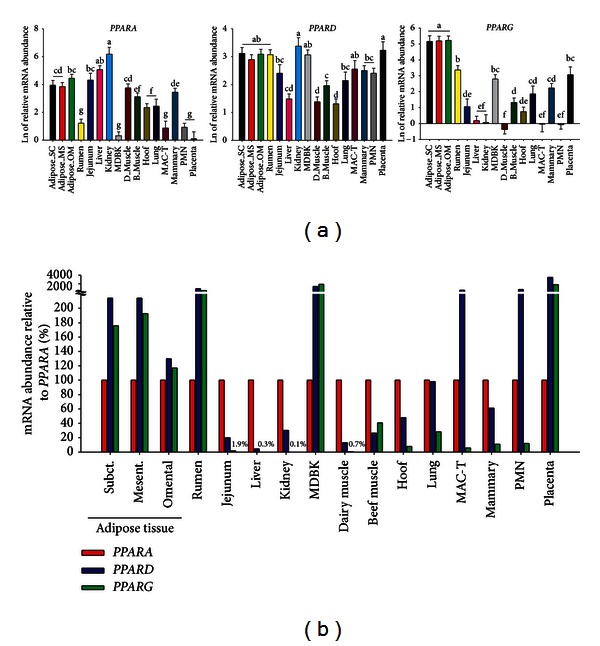
(a) Relative transcript abundance of each PPAR isotype in several bovine tissues and cells. We measured gene expression of PPAR isotypes in 14 different tissues including tissues from adult dairy cattle: adipose tissue (subcutaneous, mesenteric, and omental), small intestine (jejunum), liver, hoof corium, lung, kidney, mammary gland, blood polymorphonuclear leukocytes (PMN), and placenta; from dairy calves: rumen papillae and semitendinosus muscle (D-muscle); skeletal muscle of beef cattle (Longissimus *lombarum*); and two cell lines: Madin-Darby Bovine Kidney (MDBK) and bovine mammary alveolar cells (MAC-T). The total RNA was extracted and qPCR performed as previously described [26]. The qPCR data were normalized by the geometrical mean of 5 internal control genes (*PPP1R11*, *RPS15A*, *ACTB1*, *MRPL39*, and *UXT*). For the difference of each PPAR isotype abundance between tissues, the qPCR data were transformed using a 6-point standard curve prior statistical analysis using PROC GLM of SAS (version 9.3) with tissue as main effect. Dissimilar letters denote significant differences (*P* < 0.05). (b) Tissue-specific relative mRNA abundance between PPAR isotypes. The % relative abundance of the three PPAR isotypes in each tissue was calculated using the delta Ct method as previously described [27]. The final data for *PPARG* and *PPARD* were obtained as % relative to *PPARA*. N.B.: the *y*-axis values in (a) are least square means of the Ct values transformed using the standard curve and then log2-transformed. The values in (b) are calculated without use of a standard curve. Therefore, the values in (a) are radically different compared to the values in (b) and the two cannot be compared.

Judging from the published literature, the interest on PPAR isotypes in ruminants, particularly their role in lipid metabolism, has been modest compared to the vast literature in nonruminants, including human. Therefore, information about protein and gene expression abundance in ruminants is relatively scant. In order to help close this gap of knowledge we have performed Real-Time RT-PCR (qPCR) analysis to provide an evaluation of the relative distribution of PPAR isotypes in bovine tissues of adult Holstein dairy cows (i.e., three adipose depots, jejunum, liver, kidney, hoof corium, lung, placenta, and mammary), Holstein calves (semitendinosus muscle and rumen epithelium), longissimus muscle from Angus beef steers, and two cell lines obtained from adult bovines (Figure 1(a)). The data revealed that overall the relative distribution of PPAR isotypes in bovine tissues/cells is similar to other species.

### 3.1. PPAR*γ*

This PPAR isotype has been the most-studied in ruminants. Our results from qPCR analysis (Figure 1(a)) indicated that *PPARG* expression is very high in all adipose tissues, followed by rumen, Madin-Darby Bovine Kidney cell line (MDBK), and placenta with moderate-to-low mRNA expression in small intestine, beef cattle longissimus muscle, hoof corium, lung, and mammary gland. In contrast, the lowest expression of *PPARG* was detected in liver, kidney, dairy calf semitendinosus muscle, bovine mammary alveolar cell line (MAC-T), and blood polymorphonuclear leukocytes (PMN) (Figure 1(a)). In an early study bovine PPAR*γ* mRNA expression (via northern blot) was characterized in several tissues [29]. Similar to our data (Figure 1(a)), a greater expression of *PPARG* was detected in adipose tissue followed by spleen, lung, and ovary. Although lower, expression was also detected in mammary gland and small intestine. Expression was absent in pancreas and almost undetectable in liver. In other tissues the expression was very low or nondetectable. The *PPARG* is highly expressed in adipose tissue of mice [6], human [9], and chicken [30], all of which agree with the relative high expression in bovine adipose tissues (Figure 1(a)). Similar to mouse [6], human [9], pig [31], chicken [30], and beef bulls [32], the expression of *PPARG* in bovine liver, or other tissues such as kidney and intestine, was very low (Figure 1(a)).

We and others have previously detected expression of *PPARG* in bovine mammary tissue and the MAC-T cell line using qPCR [26, 33, 34]. In a recent study in our laboratory comparing gene expression between mammary gland and MAC-T cells, the former had greater expression of *PPARG* both during pregnancy and lactation [35]. The relatively high expression of *PPARG* in MDBK cells detected (Figure 1) confirmed previous observations [36]. Expression of *PPARG* was detected also in goat mammary, although at a significant lower level compared to bovine [37].

The *PPARG* is expressed at all stages during bovine embryo development (both in the inner mass and in the trophectoderm [38]) and in the placenta (cotyledons and caruncles) of bovine [39] and sheep [40], with an evident expression in the trophoblast [41]. Lutein cells [42] and uterus [43] express *PPARG*, but not bovine endometrial cells [44], while endometrial cells of pregnant ewes express this NR [41]. The expression of *PPARG* in ovary was confirmed in sheep [45] and the same study reported expression in pituitary gland but not hypothalamus. In previous studies it has been shown that this PPAR isotype is expressed in bovine aortic endothelial cells [46], beef cattle skeletal muscle (including intramuscular fat) [47], ovine intramuscular fat [48], bovine perimuscular preadipocytes [49], and bovine retinal pericytes [50]. In several beef cattle breeds, *PPARG* had a similar degree of expression in perirenal and omental adipose depots, followed by intramuscular fat and, in a minor quantity, in the longissimus muscle [47, 51].

The expression of various PPAR*γ* isotypes in buffalo was recently evaluated [52] and found to be expressed in all tissues tested: ovary (follicles and corpus luteum), mammary gland, adipose tissue, liver, spleen, and lung. The isoforms PPAR*γ*1a and 1b were highly expressed in ovarian tissue followed by spleen and mammary gland, respectively, while PPAR*γ*2 was highly abundant in adipose tissue.

### 3.2. PPAR*α*

This isotype has been less studied compared with PPAR*γ*. The bovine PPAR*α* gene is located in chromosome 5 in cattle [53]. The qPCR analysis of the relative mRNA abundance of *PPARA* highlighted, as in mice [6], human [54], and pig [31], that *PPARA* is very abundant in kidney (Figure 1(a)). Contrary to this general feature, even though the *PPARA* in liver of chicken is expressed at lower level than kidney, its expression in liver is similar to other tissues [30]. In contrast to what is observed in human [54], our data revealed that the relative abundance of *PPARA* was not statistically different between jejunum and adipose tissues of bovine (Figure 1(a)). In general the data in Figure 1(a) reveals a more widespread expression of this PPAR isotype among the tissues and cells evaluated compared to *PPARG*. The highest expression was observed in kidney and liver followed by adipose tissues, small intestine, and dairy cattle semitendinosus muscle. Beef cattle longissimus muscle and mammary gland had relatively modest expression of *PPARA *followed by the least expression in hoof corium, lung, rumen, MDBK, MAC-T, PMN, and placenta (Figure 1(a)). We and others have consistently detected expression of *PPARA* in liver [55–60] and in MDBK cells in which also its activity was confirmed [28, 36, 61]. Partly corroborating our data (Figure 1), this PPAR isotype has been detected in bovine endothelial cells [62], skeletal muscle [63], rumen [64], uterus [43, 65], and neutrophils [66]. Similar to our data, it was observed very recently in young Limousin bulls that *PPARA* is expressed in liver, adipose, and muscle, with the greatest expression observed in liver, followed by semitendinosus muscle, and, then, intermuscular adipose tissue [32]. In ewes, its expression was detected in superficial endometrium and trophoblast during early pregnancy [41]. Lastly, expression of *PPARA* was demonstrated in sheep heart [67].

### 3.3. PPAR*β*/*δ*

As for nonruminants, the PPAR*β*/*δ* is the least-studied PPAR isotype also in ruminants, with few published information available. The results of our qPCR analysis indicate relatively similar *PPARD* mRNA expression in all the 14 tissues and cells assessed (Figure 1(a)); however, the greatest expression was observed in kidney and placenta followed by adipose tissues, rumen, and MDBK cells with the lowest expression observed in hoof corium, liver, and skeletal muscle (Figure 1(a)). The relative distribution of *PPARD* expression among cattle tissues/cells, even though similar to that in mouse [6], is rather curious particularly considering its low expression in skeletal muscle and the marked expression in blood neutrophils, placenta, and rumen tissue, that is, tissues that probably do not rely on LCFA oxidation as source of energy. Previous studies have observed expression of *PPARD* in bovine liver [56], aortic endothelial cells [68], mammary cells [69, 70], rumen [64], and uterus [43]. The *PPARD* was also shown to be expressed in longissimusmuscle of beef steers [47] and in both superficial endometrium and trophoblast of early pregnant ewes [41].

### 3.4. Relative Abundance between PPAR Isotypes in Cattle Tissues

To date, there is almost a complete lack of data available in the literature of a direct comparison of PPAR isotypes expression in ruminant tissues. Among the few available studies, it was observed that liver of dairy cows expresses a similar amount of *PPARA* and *PPARD* but does not express *PPARG* [44]. In a recent study where the expression of the three PPAR isotypes was evaluated in liver and muscle of beef bulls, the greatest expression was observed for *PPARA*, followed by *PPARG*, with the lowest expression for *PPARD *in liver, while, the largest expression in muscle was observed for *PPARG *[71]. This relative distribution among tissues is somewhat comparable to our data (Figure 1(b)). More numerous are the studies comparing mRNA abundance between PPAR isotypes in bovine cell culture. Those have revealed that bovine endometrial cells express *PPARA* and *PPARD* at a similar level, but not *PPARG* [44]. In addition, bovine aortic endothelial cells express both *PPARA* and *PPARG* [46] and mammary cells express both *PPARG* and *PPARD* [69].

When the relative mRNA abundance between the three PPAR isotypes was evaluated in several tissues from bovine (Figure 1(b)), we observed that the three adipose tissues along with rumen, MDBK cells, and placenta have a marked abundance of *PPARD* and* PPARG* compared with *PPARA*, whereas MAC-T cells and PMN were characterized by marked abundance of *PPARD* but very low abundance of the other two PPAR isotypes. Despite the relatively low abundance, at least *in vitro*, PPAR*γ* appears to be functional in bovine neutrophils [72] and MAC-T cells [26]. Paradoxically, given its well-established function in monogastrics, with few exceptions (i.e., MDBK and beef cattle longissimus muscle), *PPARD* is more abundant than *PPARG*, even in the three adipose depots (Figure 1(b)). The *PPARA* instead was the more abundant PPAR isotype in small intestine, liver, kidney, skeletal muscle, hoof corium, lung, and mammary gland (Figure 1(b)).

Overall, the data in Figure 1 depict a distribution of PPAR isotypes that, similar to other species, seems to underscore the putative biological role of each PPAR isotype. For instance, the expression of *PPARA* is more abundant in tissues where LCFA oxidation is generally higher (e.g., liver and kidney) and *PPARG* is more abundant in lipogenic tissues (e.g., the three adipose tissues).

## 4. Sequence Homology, 3D Structure, and Activation of PPAR*α* among Bovine, Mouse, and Human

We recently carried out an *in silico* analysis to compare the amino acid sequence homology of PPAR*α* between bovine, mouse, and human [28]. The analysis revealed more than 90% conservation of this PPAR isotype between the three species, with bovine having greater overall homology to human (94.9%) than mouse (91.2%). When the four domains of the PPAR*α* protein were compared, we observed lower conservation in the N-terminal A/B domain containing the ligand-independent activation function (AF-1), which was 86% conserved between bovine and human and 81% between bovine and mouse [17], and the largest conservation (i.e., 100%) in the DNA-binding domain. The latter suggests that the capacity of the domain for the recognition of the PPRE is highly conserved between species. This has been confirmed by the high responsiveness of rat PPRE when transfected in bovine endothelial cells [73].

The LBD is also highly conserved with greater homology of bovine with human (98%) than with mouse (92%). The lower conservation of the LBD and AF-1, which is common between species, could indicate a difference in interspecies sensitivity of PPAR*α* activation [17] and a greater similarity between bovine and human than bovine and mouse. Surprisingly, when the transcription response of 30 putative PPAR*α* target genes to the potent and specific PPAR*α* agonist Wy-14643 were compared between mouse liver, human liver, and MDBK, we observed a greater number of genes with a common response between bovine and mouse (73%) than bovine and human (60%) [28]. Despite the limitation of comparing liver with kidney cells, those data indicate a good degree of conservation of PPAR*α* response between species. There are no published studies comparing PPAR*γ* or PPAR*β*/*δ* response between ruminant and nonruminant species considering the same (or similar) tissue/cells. An attempt to compare the activation of PPAR*γ* in mammary gland between dairy cattle and mouse is reported (see Section 9.2.1).

**Figure 2:**
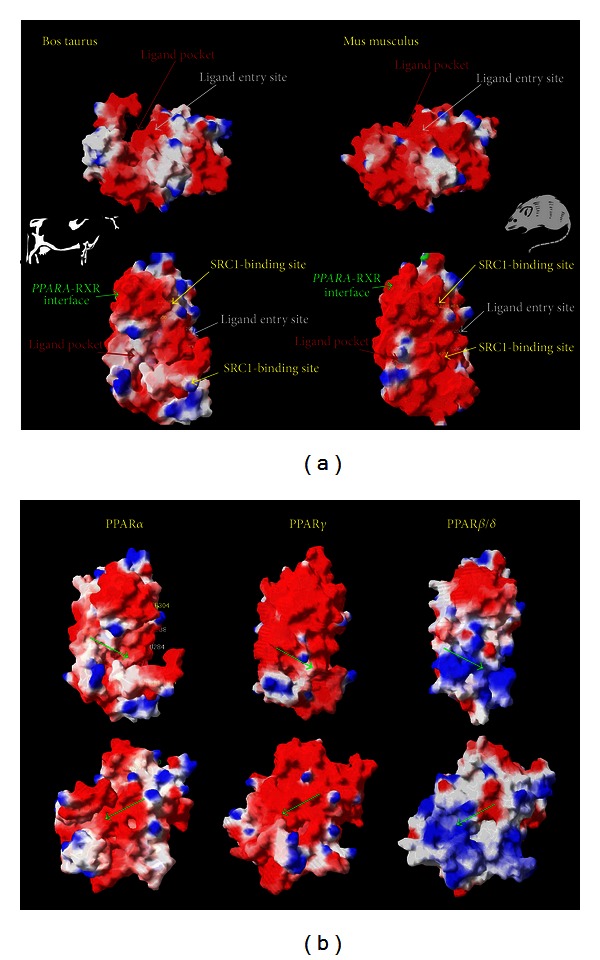
Inter-species and inter-isotypes three-dimensional PPAR protein structure comparisons. (a) Three-dimensional surface structure of bovine (residue 202–470; UniProtKB/TrEMBL Q5EA13) and mouse (residue 202–468; UniProtKB/TrEMBL P23204) PPAR*α* ligand binding domain (LBD). The upper and lower panels include two views of the 3D structure of the PPAR*α* protein in bovine and mouse species. The 3D structure is in full alignment between species. From the comparison, the difference in the ligand pocket of the PPAR*α* between the two species is evident, with a larger and more pronounced pocket in bovine compared with mouse. In addition, the bovine PPAR*α* appears to be more neutrally charged compared with the same protein in mouse. (b) Three-dimensional surface structure comparisons between PPAR*α* (residue 202–470; Q5EA13), PPAR*γ* (residue 234–505; O18971), and PPAR*β*/*δ* (residue 171–441; A4IFL4) LBD of bovine. Shown is the ligand pocket domain (green arrow) in two diverse views for each of the PPAR isotypes. The comparison highlights the larger and more neutrally charged ligand pocket in PPAR*α* compared with the more negatively charged PPAR*γ* ligand pocket and positively charged and small PPAR*β*/*δ* ligand pocket. The images were modified from [28]. Legend: red = negative charge; white = neutral charge; blue = positive charge. The 3D analyses were performed using Swiss-Pdb Viewer software (freely available at http://spdbv.vital-it.ch/).

In order to further investigate the potential differences in PPAR*α* between mouse and bovine we performed an *in silico* 3-dimensional (3D) structure analysis of the publicly available PPAR*α* protein sequence [28]. The alignment analysis identified an overall high degree of conservation of PPAR*α* amino acid sequence between the two species; however, when the overlap of the 3D structure of the PPAR*α* of the two species was performed, we observed important differences in spatial structure of the LBD. In particular, the residues Leu462 and Tyr466 of the LBD in bovine result in a completely different spatial position compared with mouse (Figure 2). When the electrostatic potential of the surface was visualized, it was apparent that the bovine PPAR*α* has an overall more neutral charge, particularly in the ligand pocket, compared with the highly negatively charged mouse PPAR*α*. This allowed inferring that longer and more saturated LCFA (i.e., more neutrally charged and with a more straight configuration) might be more easily accommodated (Figure 2), hence, likely be better inducers in bovine.

It has been demonstrated, however, that the activation of PPAR isotypes is highly dependent on the A/B domain rather than the LBD [74]. This last observation could explain the interspecies differences observed, considering also that the A/B domain is the least-conserved between species and also between PPAR isotypes (see below). However, this does not fully explain the results from the comparison in PPAR*α* response between bovine, mouse, and human [28] because the conservation of the A/B domain is lower between mouse and bovine than between human and bovine, despite the greater similarity in response between bovine and mouse compared to bovine and human [28].

## 5. Structural Similarity between PPAR Isotypes in Bovine

Approximately 80% of the 34 amino acid residues in the binding cavity of the three PPAR isotypes (*α*, *β*/*δ*, and *γ*) are conserved in humans and rodents [75]. The main features dictating the ligand specificity across the PPAR isotypes appear to be the topology of the ligand binding cavity; for example, the PPAR*β*/*δ* cavity is much narrower than PPAR*α* and PPAR*γ* and, thus, cannot accommodate bulky polar heads found in thiazolidinedione (TZD) [75, 76]. In contrast, TZD is a potent ligand of PPAR*γ*. Once inside the cavity, the side chains of the ligand (e.g., hydrogen, carboxyl groups) interact with the amino acid residues to achieve a stable configuration.

In bovine, the three PPAR isotype proteins have low conservation overall, with PPAR*α* being more similar to PPAR*β*/*δ* (59%) than PPAR*γ* (52%) [28]. The three proteins have a large degree of conservation in the DNA binding domain (>80%), but a low degree of conservation in the A/B domain (<21%) [28]. The PPAR*α* has a greater degree of conservation in the LBD with PPAR*β*/*δ* (71%) than PPAR*γ* (64%) [28]. This last observation suggests that among the three isotypes, the expected response to agonists should be more similar between PPAR*α* and PPAR*β*/*δ* as it is the case in nonruminants [2]. This would imply that activation of PPAR*α* and PPAR*β*/*δ* could result in similar outcomes, for example, fatty acid catabolism.

The 3D depiction of the bovine PPAR isotypes surface reveals a difference in the ligand pocket (Figure 2) [28]. The PPAR*α* appears to have a larger pocket compared with the other two PPAR isotypes. In addition, analysis of the electrostatic potential of the surface indicates a greater negative charge in PPAR*γ* than PPAR*α* and PPAR*β*/*δ*, with the latter being mostly positively charged. Those observations suggest a greater capacity of PPAR*α* for binding neutrally charged and/or more structurally rigid compounds. Clearly, this inference is only speculative.

## 6. Ruminant PPAR Response to Synthetic and Natural Agonists

The effect of PPAR agonists in nonruminants has been tested in different models using *in vitro* systems with specific assays such as the Coactivator-Dependent Receptor Ligand Assay (CARLA) [18] or the transfection of PPRE with firefly luciferase (e.g., [96]). An additional assay available today is the direct measurement of activation of PPAR isotypes after nuclear isolation by the presence of PPRE immobilized onto the bottom of cell culture wells; however, such assays have not been developed for ruminants [61]. The use of these techniques with greater sensitivity, precision, and reliance in ruminants has been scant [61]. Most of the studies performed in ruminants are based on measurements of changes in expression of genes or proteins after treatment with PPAR isotype-specific agonists.

### 6.1. Ruminant PPAR Response to Synthetic Agonists

Several synthetic PPAR agonists are available today for nonruminants [18]. Among the most commonly used are Wy-14643 and fenofibrate as PPAR*α* agonists and TZD and rosiglitazone as PPAR*γ* agonists. Very few synthetic agonists of PPAR*β*/*δ* are known (e.g., GW501516). Besides agonists, a few antagonists have been developed, for example, the PPAR*γ* specific antagonists GW9662 [97] and BADGE [98], the PPAR*α* antagonists T0070907 [99] and GW6471 [100], and the PPAR*β*/*δ* antagonists GSK0660 [101] and GSK3787 [102]. The use of the specific agonists in combination with antagonists could be a valid, though indirect, approach to uncover both the existence of an active PPAR isotype in cells or tissues and PPAR target genes.

Supplementary Table 1 (see Supplementary Material available online at http://dx.doi.org/10.1155/2013/684159) contains a summary of studies performed to date using specific PPAR agonists in ruminants. From the data, it is evident that most of the studies dealt with bovine with few ones in sheep and goat. A large amount of the bovine studies were performed with bovine endothelial cells. Those cells have been widely used as a model to study endothelial physiology and pathology, particularly for the inflammatory status related to arteriosclerosis, that is, with a clear biomedical purpose and not to understand ruminant biology. Overall those studies established important roles of PPAR in endothelial cells [46, 92, 103, 104]. In particular the activation of PPAR*γ* and PPAR*α* appears to have a protective role for endothelium (Supplementary Table 1).

The first study performed using a PPAR agonist with a clear aim to understand the biology of ruminants was performed in 1998 by a German group [42] where it was observed that PPAR*γ* controls progesterone synthesis in lutein cells isolated from dairy cows. Subsequent studies in granulosa cells of sheep confirmed the role of PPAR*γ* in controlling progesterone synthesis [45].

In 1998, a Japanese group demonstrated that activation of PPAR*γ* is central for adipogenic differentiation of vascular stromal cells from bovine adipose tissue [105] and intramuscular fibroblast-like cells [106]. In 2001, another Japanese group demonstrated that *in vivo* injection of the PPAR*γ* agonist 2,4-TZD partially reversed the insulin resistance induced by TNF*α* in dairy steers. The phenomenon was explained by the activation of PPAR*γ* in adipose tissue [107]. A year later a group of researchers from a pharmaceutical company fed the PPAR*α* agonist Wy-14643 to lactating goats [108]. The authors reported an overall increase in hepatic *β*-oxidation and aromatase activity by Wy-14643 and decreased cholesterol in blood (with numerical decrease of TAG as well). No effects were observed on liver size, milk composition, or content of hepatic cytochrome P450. The low magnitude of changes and the unexpected lack of effect of the treatment on P450 led the authors to conclude that the goat is a weak responder to PPAR*α* agonists.

The two studies *in vivo* mentioned above were critical for animal bioscientists interested in PPAR because they demonstrated that PPAR*α* in liver and PPAR*γ* in adipose tissue of ruminants are active and likely play similar roles as in monogastrics: regulation of *β*-oxidation for PPAR*α* and regulation of adipogenesis and insulin sensitivity for PPAR*γ*. Since then, few additional *in vivo* studies using PPAR agonists with agricultural aims have been performed (Supplementary Table 1). Recently, we tested the effects of oral administration for 14 days of the PPAR*α* agonist clofibrate on liver of weaned dairy calves [78] (see also Supplementary Table 1). The treatment had several expected effects such as the increase in expression of several PPAR*α* target genes (see Section 7 for details about PPAR targets in ruminants), but the magnitude of response was lower than usually observed in rodents; thus, we concluded, as for the work performed on goats, that the bovine hepatic PPAR*α* is a weaker responder compared to rodents.

The above observations from *in vivo* studies of a weak response in ruminants might be explained by the inherent differences in digestive physiology. Contrary to monogastrics, in ruminants, the digestion of any feed is markedly affected by the process of fermentation in the rumen via microorganisms. None of the above studies have assessed the effect of the rumen on PPAR agonists. In this regard, it could have been interesting to measure the blood concentration of the agonists. Interestingly, the human PPAR isotypes also appear to have a lower response compared with rodents [19]. It can also be the case that Wy-14643, a recognized potent PPAR*α* agonist in rodents, is not as potent in ruminants. In accord with this, we have observed in bovine cells a greater increase in expression of PPAR*α* target genes by saturated LCFA compared to Wy-14643 [28]. Those responses indicate a species-specific response to PPAR induction and a different effect of agonists between species.

The results obtained during the *in vivo* study of the Japanese group mentioned above led to a series of *in vivo* experiments in pregnant and lactating dairy cows [82, 84, 109, 110]. The purpose of those studies was to evaluate the effects of PPAR*γ* activation on preventing metabolic problems typical of the peripartal period. The specific PPAR*γ* agonist 2,4-TZD was used (via injection) for that purpose (Supplementary Table 1). The treatment with 4 mg/kg BW daily of 2,4-TZD during the last two or three weeks prepartum until parturition decreased substantially the NEFA post-partum. Such effect was ascribed to enhanced insulin sensitivity and *PPARG* expression in adipose. In addition, the treatment improved the overall metabolic health postpartum, as reflected in greater feed intake, lower hepatic lipid accumulation, and greater glycogen content in the liver. Overall, the data also suggested an improved fertility (i.e., lower open days) in cows treated with 2,4-TZD.

This series of *in vivo* experiments reported above (see also Supplementary Table 1) was the first demonstration that PPAR isoforms can play a pivotal role in the physiology and metabolism of dairy cattle. It also underscores the concrete possibility of fine-tuning the PPAR isotype activity through appropriate treatments in order to improve overall performance and health of dairy cattle.

An elegant *in vivo* study performed recently in pregnant sheep involved the injection of rosiglitazone into the fetuses for >10 days beginning at ca. 25 days before term [81]. The experiment demonstrated that activation of PPAR*γ* had a similar effect on fetuses as overnutrition of the pregnant mother, which is known to induce obesity in later life in offsprings. For instance, rosiglitazone treatment increased expression of lipoprotein lipase and adiponectin in adipose tissue and *PPARA* and PPAR*γ* coactivator 1 alpha (*PPARGC1A*) in liver of fetuses (Supplementary Table 1).

Several *in vitro* studies using synthetic agonists have demonstrated that activation of PPAR isotypes (except gamma) affects fertility by increasing the expression and/or production of prostaglandins, for example, prostaglandin (PG) F2*α*, and PGE2 in bovine endometrial cells [44, 77]. Other *in vitro* studies were carried out in order to test the response to PPAR isotypes in two bovine cell lines (MDBK and MAC-T) with the purpose of determining PPAR*α* and PPAR*γ* target genes [26, 28, 36, 61]. Besides target genes, those studies also uncovered several biological functions of PPAR isotypes in ruminants. For instance, the activation of PPAR*γ* in MAC-T cells with rosiglitazone provided a demonstration that PPAR*γ* controls expression of several genes known to be involved in milk fat synthesis [26] while activation of PPAR*α* controls lipid metabolism at the cellular and organismal level (i.e., by controlling expression of several signaling molecules) [28].

All the above studies clearly demonstrated an active role of PPAR isotypes in ruminants. The studies also established that PPAR isotypes can be manipulated by using synthetic agonists; however, from a practical stand-point the suggestion of using synthetic agonists is not feasible, namely, because of the high costs that would be incurred. Clearly that could be circumvented if natural ligands are identified.

### 6.2. Ruminant PPAR Response to Natural Agonists

#### 6.2.1. LCFA

The great interest in PPARs in the area of nutrition stems from the ability to bind and be activated (or inhibited) by LCFA or chemically related derivatives [18, 111, 112].

*Monogastrics*. In monogastrics all PPAR isotypes are sensitive to fatty acids, particularly LCFA. Although the potency varies with each PPAR isotype, the most-potent PPAR endogenous ligands in nonruminants are linoleic acid, linolenic acid, arachidonic acid, and also derivatives of arachidonic acid such as leukotriene B4 (LTB4) or PG [12]. In general it is safe to conclude that PPAR isotypes in most monogastrics species studied to date have a greater sensitivity towards unsaturated than saturated [17, 18]. However, in nonruminants both saturated and unsaturated LCFA enhance PPAR transactivation *in vitro* (e.g., [12, 113, 114]).

*In vivo* data have been more variable and in some instances high dietary fat activated PPAR target genes regardless of whether the dietary lipid was mostly polyunsaturated (PUFA), monounsaturated, or saturated (e.g., [115]). At the cellular level studies with endogenous ligands such as free LCFA or LCFA-CoA (i.e., activated 16:0, 18:2n-6, 18:3n-3, and 20:4n-6) have demonstrated (at least for PPAR*α*) that both forms of the FA exhibit high affinity (i.e., low nanomolar dissociation values) for the ligand-binding domain of PPAR [114]. This point is important because intranuclear concentrations of free LCFA and LCFA-CoA range between 120–500 nM and 8 nM, respectively [116].

From a mechanistic standpoint it is important to point out that FA binding proteins (FABP, particularly FABP1 and FABP4) are important in channeling intracellular nonactivated (i.e., without addition of the CoA group) LCFA not only to the various organelles but also to the nucleus where the LCFA can activate PPAR. The essential role of FABP in transporting LCFA into the nucleus for the activation of PPAR isotypes was first reported in rodent liver where the amount of FABP1 protein significantly correlated with transactivation of PPAR in response to LCFA (linoleic acid, linolenic acid, and arachidonic acid) as well as other chemical ligands [117].

*Ruminants*. To our knowledge there are only two published studies where PPRE luciferase was used to test activation of PPAR isotypes in bovine cells [62, 68]. In one study, however, only activation of PPAR*β*/*δ* was assessed and no LCFA were tested. In another study the activation of PPAR*α* by free LCFA or oleic acid was demonstrated in bovine aortic endothelial cells [62]. So far the effect of LCFA on ruminant PPAR activity has been evaluated primarily in an indirect way through measuring changes in expression of target genes after addition of specific LCFA. This model has limitations, one being the capacity of LCFA to bind and activate additional transcription factors (TF). Besides PPARs, also Hepatic Nuclear Factor 4 (HNF4*α*), Liver X Receptor (LXR), and RXR can bind LCFA, as shown in human, mouse, and rat [118]; however, in those species the LXR*β* and the RXR*α* appear to be weakly activated by natural LCFA while PPAR*α*, PPAR*β*/*δ*, and PPAR*γ* are strongly activated [119]. The greater sensitivity of PPAR compared with other TF provides some support for the use of target gene expression as a proxy for evaluating activation of PPARs by LCFA. Another limitation of the indirect approach is the inability to distinguish the activation between PPAR isotypes. Using the above indirect approach it was demonstrated that ruminant PPAR are activated by several physiologically relevant LCFA (Table 1).

The LCFA experiments in ruminants were mainly performed with MAC-T and MDBK cells and focused on PPAR*α* and PPAR*γ* [26, 28, 36, 61]. In both cell types the LCFA clearly induced expression of genes previously shown using specific agonists (Wy-14643 and rosiglitazone) to be PPAR*α* and PPAR*γ* target genes (see Table 2 and Section 7 for details). The potency of saturated was greater than unsaturated LCFA. In particular, in MDBK cells we observed weaker induction of target genes as the degree of unsaturation increased [28]. Above all it was observed that palmitate and stearate induced a very strong activation of transcription of PPAR*α* and PPAR*γ* target genes [26, 28]. Those data were suggestive of an evolutionary adaptation of the PPAR in ruminants to respond to saturated LCFA, which are the most abundant LCFA in the circulation of ruminants [120, 121] compared to monogastrics [122, 123] due to extensive ruminal hydrogenation of unsaturated LCFA. However, our studies suggested that the LCFA activated gene expression not only through PPAR isotypes but also other TF, probably the ones mentioned above, or even other unknown TF [28]. This point, as well as the role of coactivators and their relative abundance [76], deserves further investigation in order to select with greater confidence the most suitable mixture of LCFA for modulating metabolism in ruminants.

Because intracellular LCFA pools are a mixture of saturated and unsaturated LCFA, it is interesting that PPAR*γ* (and maybe other PPAR isotypes) is capable of binding two LCFA simultaneously, at the least in monogastrics [124]. This suggests that there could exist a mechanism whereby the composition of LCFA in the cytosol dictates the “strength” of the response, that is, the ability to bind two LCFA simultaneously could allow PPAR*γ* to give a graded response to the varying composition of the intracellular LCFA pool [124].

#### 6.2.2. Glucose

Besides LCFA, it has been also reported that glucose binds and activates PPAR*α* in mouse connecting glucose with lipid metabolism [125]. This has not been confirmed in ruminants; however, it has been shown that ruminant PPAR*β*/*δ* binds and is activated by glucose [68]. Specifically, it was demonstrated in bovine endothelial cells that when PPAR*β*/*δ* is activated by glucose, it downregulates glucose transport in order to prevent hyperglycemia.

#### 6.2.3. Other Natural Agonists/Antagonists

As with nonruminants, PPAR*γ* in bovine vascular endothelial and mammary cells is activated by PGJ2 [46, 69]. The PPAR*γ* is inhibited and its expression decreased by the oxidative stress intermediate H_2_O_2_ in bovine endothelial cells [94, 126]. Nitric oxide appears to be an inhibitor because it decreased the expression of the *PPARGC1A*, a known PPAR*γ* target gene [94]. This compound decreased the expression of *PPARGC1A* during the first 12 h after treatment but increased the expression of the same gene in the longer term (>24 h) [127]. The increase in expression of *PPARGC1A* was demonstrated to be crucial for the mechanism of protection from oxidative stress [127]. In bovine articular chondrocytes, the presence of oxidized LDL increased expression of vascular endothelial growth factor (VEGF) through PPAR*γ* [128].

## 7. PPAR Isotype Target Genes in Ruminants

In several of our studies, the overall response of PPAR*α* and PPAR*γ* in bovine cells was strong and consistent [26, 28, 61, 129]. Those studies allowed uncovering several bovine-specific PPAR*α* target genes (Table 2), and several were already established as PPAR*α* targets in other species. Among bovine-specific PPAR*α* target genes, the osteopontin (*SPP1*) gene had a large increase in expression after Wy-14643 treatment in bovine kidney cells [28] contrary to what has been observed in human and mouse [130, 131]. Between bovine, human, and mouse, only 67% of the putative PPAR*α* target genes tested responded in a similar fashion, suggesting a species-specific response of PPAR [28].

The activation of PPAR*α* by Wy-14643 resulted in a general increase in lipid metabolism-related genes including several involved in lipid synthesis, such as lipin 1 (*LPIN1*) and sterol regulatory element binding transcription factor 1 (*SREBF1*) [28]. Interestingly, expression of both genes was not induced in a previous study using the same model [61]. The only difference between the two studies was the addition of insulin in the latter [28]. In support of a potentially important role of insulin for PPAR activation, in a recent study with MDBK, we observed a faster response in expression of PPAR*α* target genes after addition of insulin [61]. Therefore, insulin in bovine seems essential for PPAR activation but may be more crucial for some genes (e.g., *LPIN1* and *SREBF1* versus carnitine palmitoyltransferase 1A (*CPT1A*)) [28, 61].

The increased expression of *SREBF1* with Wy-14643 in the MDBK study might also be due to the activation of PPAR*γ* because we observed that activation of PPAR*γ* with rosiglitazone increased expression of *SREBF1 *in MAC-T cells [26]. The activation of PPAR*γ* in MAC-T cells appeared to be robust [26]; however, the use of 10 *μ*M TZD for 12 h in MDBK cells did not affect expression of any gene tested using microarray technology, suggesting that activity of PPAR*γ* in MDBK is extremely low or inexistent (Bionaz et al. unpublished data). This observation is intriguing considering that overall expression of *PPARG* in MDBK is relatively high compared with other tissues/cells (Figure 1(a)), and higher than *PPARA* (Figure 1(b)). Furthermore, the response to PPAR*α* agonists is consistently high in those cells [28]. Therefore, it cannot be excluded that the increase in expression of *SREBF1* after addition of Wy-14643 was due exclusively to PPAR*α* activation.

Compiled data from our and other groups in Table 2 suggest that there are some inconsistencies in the response of target genes between tissues or cells, or even between the same tissue/cell. This is not surprising considering that several conditions can change the activity of PPAR isotypes, for example, the addition of insulin mentioned above. However, another important factor that might explain the different response between cell types or experiments is the abundance and activity of coregulators [132].

Some unexpected findings can be seen from data reported in Table 2. For instance, the well-established PPAR*γ* target in nonruminants *FABP4* [133] does not appear to be affected by activation of PPAR*γ* in ruminants, at least in MAC-T cells [26] but was induced by activation of PPAR*α* in MDBK cells [28]. In a study performed in intramuscular fat of growing beef steers, it was observed a very high correlation between the expression of *FABP4* and *PPARG* suggesting a dependence of *FABP4* expression from PPAR*γ* [80]. Contrary to such observation, in a recent study in pregnant overfed versus normal fed energy dairy cows, no change in expression of *FABP4* was observed but a greater expression of *PPARG* in subcutaneous adipose [134]. As for others, this unexpected finding in ruminant cells needs to be further confirmed; however, it underscores the limitation of using nonruminant data in the context of bovine.

Another cause of discrepancy might be due to methodological differences between studies, such as the methods used to perform qPCR. Most of the target genes reported in Table 2 were uncovered using qPCR. This technique relies on the identification and use of proper internal control genes [135], which is seldom conducted. As a result, some of the data generated by qPCR may lack accuracy prompting for a more routine application of all quality controls. In order to overcome several of the critical limitations often found in work reporting qPCR data, the minimum information for Publication of quantitative Real-Time PCR experiments (MIQE) [136] was created. Adherence to those guidelines will help standardize protocols, thus, enhancing data reliability. The use of such guidelines should be required by a greater number of scientific journals.

## 8. Effect of NEFA, Energy in the Diet and Fetal Reprogramming, on PPAR Isotypes

### 8.1. NEFA

The provision of LCFA to mammalian cells is from NEFA originating from adipose tissue lipolysis or from lipolysis of chylomicron or very low density lipoproteins (VLDL). The activation of bovine PPAR*α* by NEFA was demonstrated recently in bovine aortic endothelial cells, where it was observed that PPAR*α* activity was increased by release of free FA from VLDL via the action of lipoprotein lipase (LPL) [62]. In the same experiment it was demonstrated that ~10 *μ*M of released NEFA in the media activated PPAR*α* by ca. 80% compared to 10 *μ*M of the specific PPAR*α* agonist Wy-14643. A similar concentration of oleic acid alone activated bovine PPAR*α* up to ca. 60% compared to Wy-14643. The activation of PPAR*α* was due to free FA uptaken by the cells as demonstrated by the strong linear relationship between activation of PPAR*α* and uptake of LCFA [62]. In addition, the activation of PPAR*α* was proportionally inhibited by amount of albumin in the medium [62]. The results from the same study also indicated that the free FA released by the LPL, and not the circulating plasma FA (i.e., albumin-bounded NEFA), are the ones able to activate PPAR*α*. The authors explained this by proposing that the high concentration of LCFA needed for PPAR*α* activation can be achieved only by local release by lipase of LCFA from lipoproteins. Those results need to be further confirmed because of their important implications in the fine-tune activation of PPARs by dietary approaches.

The activation of PPAR by FA entering the cells via the unsaturable process is supported by the fact that endogenous activation of PPAR*α* *in vivo* seems to occur mainly with high levels of LCFA that occur under fasting conditions in nonruminants [137]. In addition, we have shown in bovine cells that the expression of PPAR*α* target genes is faster and more pronounced if cells are treated with free palmitate instead of palmitate bound to albumin [61].

The above-mentioned findings are relevant to dairy cattle soon after parturition when the hypoinsulinemia due to negative energy balance (NEB) reduces insulin sensitivity, and uncoupling of the growth hormone-insulin-like growth factor-1 axis results in substantial increase in NEFA, a mixture of LCFA whose composition can be partly altered through dietary approaches.

Evidence of increased activation and/or expression of PPARs due to the surge in NEFA has been reported in cattle. In particular, it has been observed that during the transition from pregnancy to lactation, characterized by a large surge of plasma NEFA, there is upregulation in expression of several PPAR target genes (e.g., *CPT1A*, *ACOX1*, see Table 2) in liver of dairy cattle, with a concomitant increase in expression of *PPARA* [57, 138, 139]; however, not all the studies found this to be a consistent response [140].

### 8.2. Nutrient Restriction

Nutrient restriction in dairy cows, causing a concomitant increase in blood NEFA, enhanced expression of *PPARA* and *PPARD* in liver [56] and protein expression of PPAR*γ* in the hypothalamus [141]. Similarly, a 60-day period of body weight loss in beef cows was associated with greater expression of all three PPAR isotypes in biceps femoris muscle and several PPAR target genes, compared with cows that maintained body weight [142]. Overall, the data indicated that the NEB, with a consequent increase in NEFA, appears to induce expression and activation of all PPAR isotypes, but particularly of *PPARA* and *PPARD*.

### 8.3. High Dietary Energy

High dietary energy during pregnancy in dairy cows was associated with lower expression of liver *PPARA* early post-partum [143]. High dietary energy in weaned Angus steers, but not Angus × Simmental steers, was associated with lower expression of *PPARD* in Longissimus* lumborum *muscle [47].

### 8.4. Dietary Energy and Fetal Reprogramming

In ovine, nutrient restriction in ewes during early pregnancy (between 28 to 80 days gestation) increased expression of *PPARA* in the adipose tissue of the near-term fetus [144]. However, this was true only if the ewes were fed to requirements after this period of pregnancy; the adipose tissue of fetuses from ewes fed ad libitum from 80 days of pregnancy to term had lower *PPARA* expression [144]. The above data clearly indicate that level of energy in the diet of the mother has a strong effect on the fetal transcriptome, that is, fetal reprogramming.

The fetal reprogramming of PPAR due to dietary energy level also has been observed when animals were overfed energy during pregnancy, such that fetuses of those dams had greater expression of *PPARG *and other lipogenic genes [145]. In contrast, either control or a high-energy diet in the periconception period or during pregnancy did not affect expression of *PPARG* in perirenal, omental, or subcutaneous adipose tissue of 4-month-old lambs [146]. Interestingly, intrafetal administration of a PPAR*γ* agonist, rosiglitazone, increased expression of *LPL*, a putative PPAR*γ* target gene, in perirenal adipose tissue of sheep fetuses [81]. No effect was observed for *PPARG* itself. In contrast, in the same study rosiglitazone increased expression of *PPARA* in liver.

## 9. Biological Effects of PPAR Activation in Ruminants

Most of the biological roles of PPAR uncovered in monogastrics can likely be extrapolated to ruminants; however, before those roles can be considered established also in ruminants, experiments need to be performed. Due to the modest amount of research performed to date, the biological significance of PPAR isotypes in ruminants is not well established, but the studies so far conducted have confirmed the existence of conserved roles between monogastrics and ruminants. In this section we provide an overview of the biological roles suggested by most of the experiments on PPAR carried out in ruminants besides those mentioned above on bovine endothelial cells.

### 9.1. Control of Adipogenesis and Lipid Metabolism

#### 9.1.1. PPAR*γ*

As for nonruminants [21], PPAR*γ* plays a pivotal role in adipogenesis in ovine and bovine [91, 147], and in dairy cows its expression is high in adipose tissue (Figure 1) and appears to control lipogenesis by acutely responding to energy level in the diet [82, 134, 148, 149]. The importance of PPAR*γ* in adipogenesis has been highlighted also by the identification of this as one of the candidate genes related to bovine marbling [150]. Besides lipogenesis, PPAR*γ* might also play a role in LCFA oxidation as recently observed in lamb pulmonary arterial endothelial cells [85]. In that study it was demonstrated that PPAR*γ* controls the expression of carnitine palmitoyltransferase 2 (*CPT2*) and carnitine O-acetyltransferase (*CRAT*), both genes involved in the entry of LCFA into the mitochondria, while it controls the translation of *CPT1A* but not its expression [85].

#### 9.1.2. PPAR*α*

The activation of goat PPAR*α* *in vivo* increased fatty acid oxidation in liver [108]. The oral administration of Wy-14643 increased palmitate oxidation in liver of dairy calves with a concomitant increase in expression of several genes known to be PPAR*α* targets (see Table 2) involved in FA oxidation in nonruminants [78]. Therefore, it is apparent that the activation of PPAR*α* in ruminants controls catabolism of fatty acids. Other pieces of evidence supporting that conclusion include the fact that FA catabolism in mitochondria and peroxisome increases during the transition from pregnancy into lactation [151]. This appears to be consequence of the large surge of NEFA and the concomitant increase in expression of few key genes rather than an increase of overall pathway flux [152]. However, the expression of *PPARA* in liver of dairy cattle increases from pregnancy to early post-partum [57, 138]. In the same time, several PPAR*α* target genes involved in lipid metabolism have a similar increase in expression as *PPARA* in liver during the transition from pregnancy to lactation; those include *ACOX1* and acyl-coenzyme A dehydrogenase, medium chain (*ACADM*) [57, 138]. Finally, the use of Wy-14643 in MDBK cells increased expression of several genes involved in lipid catabolism [28, 61] (Supplementary Table 1). One of those key genes is the well-known PPAR*α* target *CPT1A *[57, 138].

#### 9.1.3. PPAR*β*/*δ*

Compared with PPAR*α* and PPAR*γ*, the role on lipid metabolism of PPAR*β*/*δ* activation in ruminants is less clear. The PPAR*β*/*δ* was shown to have a role in adipogenesis in sheep because its activation increased activity of GAPDH [91]. An involvement of PPAR*β*/*δ* in adipogenesis also was reported by several experiments performed in monogastrics [2]. However, a contrasting role of PPAR*γ* and PPAR*β*/*δ* was observed in primary bovine mammary cells, where several PPAR*γ* ligands reduced the expression of PPAR*β*/*δ* [69]. PPAR*α* unarguably has a primary role in controlling fatty acid oxidation in rodents; however, PPAR*β*/*δ* also controls fatty acid oxidation in skeletal muscle, heart, and brown and white adipose tissue [2]. Several data indirectly suggest a similar role in ruminants. It was observed that during nutrient restriction [56] and during body weight loss in muscle of beef cows [142], both situations that enhance LCFA oxidation, there was a concomitant increase in expression of *PPARA* and *PPARD*.

In summary, the pivotal role of PPAR*γ* in controlling adipogenesis and lipogenesis in adipose tissue, which was clearly established in nonruminants, can also be considered established in ruminants. The control of fatty acid oxidation by PPAR*α* in ruminants appears supported by the data published to date. The few data available also suggest a role for PPAR*β*/*δ* in lipid catabolism in ruminants.

### 9.2. Control of Milk Fat Synthesis by PPAR*γ* in Dairy Cattle

Milk fat synthesis in dairy cows appears to be controlled at least in part by PPAR*γ*. This was originally suggested by the increase in expression of *PPARG* in mammary gland of dairy cows between pregnancy and lactation [33]. In the same study, a large increase in expression of a network of genes potentially involved in milk fat synthesis and for the most part putative PPAR*γ* target genes was observed. Based, on those data we then tested, and demonstrated, the hypothesis that PPAR*γ* controls expression of key genes involved in milk fat synthesis, including *SREBF1* [26].

A pivotal role of milk fat synthesis regulation by SREBP1 has been originally proposed based on the consistent reduction of *SREBF1* expression by *t10,c12*-CLA, a minor unsaturated FA produced during ruminal biohydrogenation of long-chain polyunsaturated FA [153]. The activity of SREBP1 is largely due to its abundance, which is controlled by the transcription and posttranscriptional regulation, and abundance and activation of the cofactors SREBP cleavage-activating protein (*SCAP*) and insulin induced gene 1 and 2 (*INSIG1* and* INSIG2*) [2, 33]. The INSIGs protein blocks SREBP1 activity when the level of oxysterol is high (see references in [33]). The reduced activity of SREBP1 by *t10,c12*-CLA is also controlled at the posttranslational level [2], but in this regard it is interesting that *t10,c12*-CLA consistently decreases the expression of *SREBF1*. Considering the unidirectional response of SREBP1 to *t10,c12*-CLA (i.e., inhibition of milk fat synthesis), and the inability of this TF to bind and be activated by other LCFA, it appears obvious that other TF must be involved in the positive response of milk fat synthesis to LCFA. Hence, it is remarkable that the activation of PPAR*γ* by rosiglitazone in MAC-T cells was accompanied by a significant increase in expression of *SREBF1*, demonstrating that *SREBF1* is a PPAR*γ* target gene in ruminants [26]. Our overall data [26, 33] suggest a concerted action of SREBP1 and PPAR*γ* in controlling milk fat synthesis but underscore a more fundamental role of PPAR*γ*, the only one among the two that is able to be activated by LCFA.

The evidence supporting a role of PPAR*γ* in controlling milk fat synthesis has recently been dismissed [153] using three different arguments; here we briefly outline those arguments and present the counterarguments.

1. The ca. 2-fold increase in expression of *PPARG* in bovine mammary gland from pregnancy to lactation [33] was interpreted as “related to differentiation and the initiation of milk synthesis rather than the regulation of milk fat synthesis during established lactation” [153]. The PPAR*γ* is known to be involved in differentiation, but almost exclusively of the adipose tissue where it plays an essential role [21, 154]. For the rest, it is known that PPAR*γ* has a negligible role in the differentiation of epidermis, one among several epithelial tissues [155]; however, a role for this PPAR isotype in differentiation of sebaceous gland after skin injury has been reported [15]. Although a role for PPAR*γ* in the differentiation of mammary gland cells cannot be fully discarded, it has not yet been reported.
2. The authors based their conclusions on the fact that CLA are activators of PPAR*γ* in monogastrics, *de facto* disregarding the findings showing that ruminant PPAR*γ* does not seem to be activated by CLA, especially in mammary epithelial cells [26] (see Table 1).
3. The most critical misinterpretation dealt with the observed increase in expression of genes related to milk fat synthesis in MAC-T cells after treatment with the PPAR*γ* agonist rosiglitazone [26]. The data clearly pointed to an active role of PPAR*γ* in controlling milk fat synthesis. The authors, using the above argument about activation of PPAR*γ* by CLA, interpreted those data exclusively from a milk fat depression angle; that is, activation of PPAR*γ* by CLA should be responsible for depressing milk fat synthesis. That was neither what the data suggested nor our conclusions [26].

In an *in vivo* experiment the activation of PPAR*γ* prepartum by TZD affected adipose tissue post-partum but, apparently in contrast to the above data, decreased milk fat production [109]. This result is not completely surprising considering that the TZD treatment was provided pre-partum when there is a large abundance of PPAR*γ* in adipose tissue and a low abundance in mammary gland [33], whereas, when PPAR*γ* is expected to increase in mammary gland due to the onset of lactation [33], the TZD was no longer supplemented and the amount of NEFA, which could have played a role in activating PPAR*γ*, was decreased in cows treated with TZD [109]. In addition, the adipose tissue competes with mammary gland for lipogenic substrates, especially if the insulin sensitivity is high, as demonstrated by the reduced milk fat by injection of insulin in cows [156]. From this point of view it would be interesting to test the effect of TZD injection post-partum on milk fat synthesis in dairy cows.

Besides PPAR*γ* and SREBP1, data from another laboratory suggested that LXR also plays a role in controlling *de novo* FA synthesis [157]. It is recognized that in order to demonstrate the central role of PPAR*γ*, SREBP1, LXR, or their combination in controlling milk fat synthesis in dairy cows, there is need for more fundamental studies, for instance, via gene-specific knock-outs. Recently, two studies from the same laboratories [158, 159] used siRNA specific for *SREBF1 *in order to define the role on controlling milk fat synthesis of this transcription factor. From the studies it was shown that basal transcription of genes involved in *de novo* FA synthesis in bovine mammary epithelium is partly under control of SREBP1. Some of the same genes were induced when LXR was activated using a specific agonist. Studies using siRNA specific for *PPARG* in bovine mammary cells are lacking. In the context of milk fat synthesis regulation, we deem more relevant the unbiased discovery of the role of LCFA in affecting the transcriptome by binding specific TF than demonstrating a more crucial role of one or another TF.

#### 9.2.1. Is PPAR*γ* Crucial for Milk Fat Synthesis Also in Mouse?

**Figure 3:**
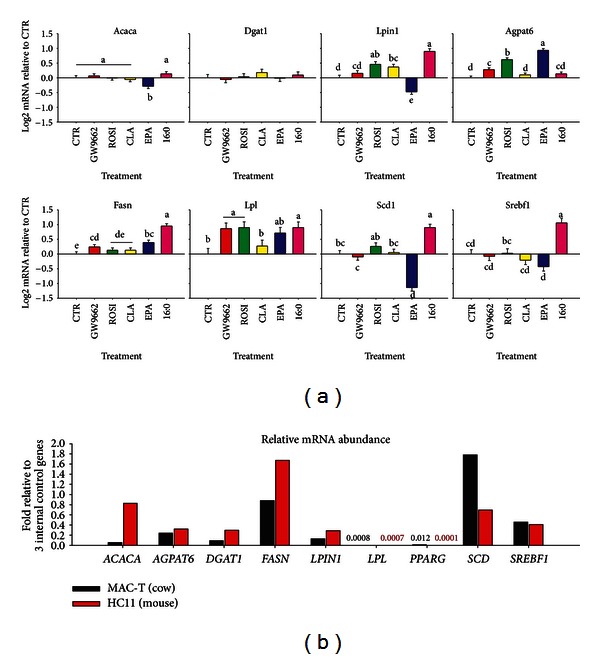
Effect of PPAR*γ* activation on genes coding for proteins involved in milk fat synthesis in mouse mammary epithelial cells HC11. The experiment was performed with the purpose to test the effects of 50 *μ*M of the PPAR*γ* activator rosiglitazone, the PPAR*γ* inhibitor GW9662, or 100 *μ*M of several long-chain fatty acids (*trans*-10,*cis* 12-conjugated linoleic acid (CLA), eicosapentaenoic acid (EPA), or palmitate (16:0)) for 12 hours in HC11 cells and compare the data with results using the same experimental design (except the GW9662 treatment) in MAC-T cells [26]. All the procedures with few modifications were as previously described [26]. The RNA was extracted and qPCR performed for several genes known to be involved in milk fat synthesis and significantly upregulated by rosiglitazone in MAC-T cells and the same 3 internal control genes used [26]. In (a), the effect of treatments on HC11 cell is reported. For that experiment, the qPCR data were calculated as fold change relative to control and log2 transformed prior statistical analysis using Proc GLM of SAS with treatment as main effect and replicate as random. Dissimilar letters denote significant differences between treatments (*P* < 0.05). In (b), a comparison in mRNA abundance between measured genes in the control group of HC11 and MAC-T cells is presented. The relative mRNA abundance was calculated as previously described [26] but as fold difference relative to the geometric mean of the median Ct values of the 3 internal control genes instead as % relative abundance. The same analysis was performed for the MAC-T cells using data previously published [26]. The *PPARG* was detectable only for few samples in HC11 cells and *LPL* was barely detectable in both HC11 and MAC-T cells.

Contrary to dairy cows [33], in mouse the mammary *PPARG* expression decreased between pregnancy to lactation [160], also after accounting for the large disappearance of adipose tissue [161]. In porcine mammary gland, the *PPARG* was not affected by lactation [162]. The expression of *PPARG* in mouse and pig mammary gland suggests that PPAR*γ* likely does not control milk fat synthesis in monogastrics. In order to further study the role of PPAR*γ* on milk fat synthesis in monogastrics, we have performed an *in vitro* experiment in mouse mammary epithelial cells (HC11; Figure 3). The experiment also was performed with the purpose of comparing the data previously generated with bovine mammary cells [26]. For this reason, the experiment was performed in HC11 with the same experimental design as the one previously performed in MAC-T cells [26]. Most of the treatments in HC11 were the same as in MAC-T cells with the exception of the PPAR*γ* inhibitor GW9662.

As observed in MAC-T cells, the saturated LCFA palmitate increased expression of several lipogenic genes in HC11 but, differently than in MAC-T cells [26], the effect appeared to be PPAR*γ*-independent due to the extremely low expression and activity of PPAR*γ* (Figure 3(a)). Those findings are intriguing because, together with the greater abundance of *PPARA* compared with *PPARG* in MAC-T cells (Figure 1(b)), suggests that the observed increase in mammary lipogenic genes due to palmitate are via PPAR*α* or other TF rather than PPAR*γ* in immortalized mammary cells from cattle and mouse.

Contrary to what was observed in MAC-T cells [26] and *in vivo* in mouse mammary gland [163], the *t10,c12*-CLA failed to inhibit the expression of lipogenic genes in HC11 (Figure 3(a)). This observation is surprising considering that the *Srebp1* expression is relatively high and with similar level in HC11 compared with MAC-T cells (Figure 3(b)). Only EPA decreased expression of few lipogenic genes in HC11; among those the *SCD* was downregulated by EPA also in MAC-T cells [26]. The relative abundance of genes measured in HC11 compared to MAC-T cells (Figure 3(b)) revealed that lipogenic gene expression is overall greater in HC11 than MAC-T, with exception of *SCD* that is more abundant in MAC-T cells. The *PPARG* had low expression in both cell lines but was virtually absent in HC11, while clearly detectable in MAC-T cells. This observation likely accounted for the fact that the PPAR*γ* agonist rosiglitazone and the inhibitor GW9662 had little effect on the expression of most genes in HC11 (Figure 3(a)). On the contrary, rosiglitazone increased the expression of all those genes in MAC-T cells [26].

The virtual absence of *Pparg* expression in HC11 (Figure 3(b)) together with the lack of decrease in expression of milk fat-related genes by CLA despite the large expression of *SREBF1* seems to indicate a role of PPAR*γ*, and more likely PPAR*γ*-SREBP1 crosstalk, in translating the lipogenic inhibition, and particularly milk fat depression effect, of CLA (and likely EPA) usually observed *in vivo*. However, the data also point to a more complex nutrigenomics response to LCFA, likely involving additional TF besides SREBP1 and PPAR*γ*.

Overall, the comparison between the mouse and the bovine mammary epithelial cell lines, with all the limitations of *in vitro* experiments, highlights a crucial difference between rodents and bovine in the genomic control of milk fat synthesis. The data clearly uncovered no roles for PPAR*γ* in controlling milk fat synthesis in mouse. Those observations suggest caution when inferring physiological responses using data from a different species.

### 9.3. Control of Inflammatory Response

The activation of PPAR*γ*, PPAR*α*, and PPAR*β*/*δ* has anti-inflammatory effects in nonruminants [19, 164] and some data are available in ruminants suggesting a similar effect. The first demonstration that PPAR*γ* might play an anti-inflammatory role in ruminants was carried out by a Japanese group by injecting for 9 days human recombinant TNF*α* plus TZD in dairy steers. They observed that the TZD treatment partially reversed the insulin resistance caused by TNF*α* [107]. The TZD effect was probably due to enhanced insulin signaling through PPAR*γ* activation by also counteracting the effect of TNF*α* [165]. The anti-inflammatory effect of PPAR*γ* in ruminants is elicited not only by counteracting the effect of TNF*α*, but also by reducing the production of this cytokine. This was demonstrated recently when treatment of bovine peripheral blood mononuclear cells with 100 *μ*M of *t10,c12*-CLA or 10 *μ*M of rosiglitazone attenuated the production of TNF*α* *in vitro*, with a stronger effect observed in cells treated with rosiglitazone [166].

In bovine primary mammary epithelial cells (bMEC), the activation of PPAR*γ* by several agonists caused downregulation of several proinflammatory cytokines and increased expression of the chemokine *CCL2* and TNF*α* [69]. In contrast, PGJ2 enhanced markedly the expression of both interleukin 8 (*IL8*) and chemokine (C-X-C motif) ligand 6 (*CXCL6*) and had no effect on other cytokines [69]. The same study also demonstrated that the generation of proinflammatory mediators in bMEC treated with lipopolysaccharide (LPS) can be modulated by synthetic PPAR*γ* agonists. These findings support a role of PPAR*γ* in mastitis resistance in dairy cows.

Some additional evidences support an anti-inflammatory role of PPAR in ruminants. The activation of PPAR*α* has shown to limit leukocyte adhesion to the bovine endothelium [167]. The expression of *PPARG* is reduced by intramammary infection with *Escherichia coli* [168] and PPAR signaling was evidently inhibited by intramammary infection with *Streptococcus uberis* [169]. The *PPARG* and *PPARA* were also markedly downregulated in PMN soon after an inflammatory challenge; however, the expression of *PPARD* increased markedly and was substantially more abundant than the other isotypes (Moyes et al. unpublished data). In contrast, the expression of *PPARA* and *PPARG* in liver was not affected after intramammary treatment with *Escherichia coli* that induced a strong hepatic acute-phase reaction [170]; however, the most-impacted biological effect of the treatment was the reduction of lipid metabolism in the liver, particularly steroid synthesis and PPAR signaling [171]. The involvement of PPAR*β*/*δ* in the process of inflammation was recently underscored when an intramammary infusion of LPS led to marked upregulation of *PPARD* and several proinflammatory genes in liver of dairy cows (e.g., *TNF*, *NFKB1*) [172].

The potential role of PPAR isotypes on inflammation can also be inferred by the fact that the expression of the PPAR*α* agonist *ANGPTL4* (Table 2) increases markedly in response to inflammation not only in mouse liver [173] but also in bovine liver [172], and it has been proposed to serve as a positive acute phase protein (+APP) [173]. In that context, it is interesting that the expression of *ANGPTL4* in adipose tissue increases markedly after parturition [134, 174], when the animals experience inflammatory-like conditions [175, 176]. Whether the upregulation of *ANGPTL4* in adipose tissue after parturition denotes a response of the tissue to an inflammatory state remains to be determined; however, there is evidence of activation of immune-related pathways in adipose tissue soon after parturition [177].

### 9.4. Control of Intertissue Metabolic Adaptations during Changes in Nutritional Status and Physiological State

In monogastrics, the PPAR*α* targets angiopoietin-like 4 (*ANGPTL4*) [178] and fibroblast growth factor 21 (*FGF21*) [179, 180] have been identified as extra-hepatic signals (hepatokines) that play an important role in the coordination of tissue adaptations to fasting, undernutrition, and the transition into lactation in bovine [56, 95, 174]. Although direct proof of bovine PPAR*α* activation as the trigger for the marked upregulation of liver *FGF21* after parturition [86, 95] is not available, the fact that the upregulation of *FGF21* was observed in animals with greater NEFA [86] is suggestive of *FGF21* as a PPAR*α* target in bovine. The link between PPAR*α* activation and *ANGPTL4* was previously discussed with data from cows suffering from undernutrition-driven ketosis [56] and was partly confirmed *in vitro* [28]. However, it was recently observed that hepatic *ANGPTL4* and *PPARD* (not *PPARA*) expression was upregulated during acute inflammation suggesting that in bovine this PPAR isotype also may regulate expression of the hepatokine [172]. Specific molecular work would need to be carried out to clarify the validity of the observed relationship in terms of a functional link.

### 9.5. Other Roles

The use of PPAR*γ* agonists decreases protein synthesis, but as demonstrated in bovine aortic endothelial cells, the mechanism appears to be independent of PPAR*γ* [181]. As with nonruminants, the activation of PPAR*γ* improves insulin sensitivity in dairy cows [82]. The activation of PPAR*α* in the liver might also increase gluconeogenesis. This was inferred by the impaired gluconeogenesis in PPAR*α*-null mice [182]; however, none of the main enzymes involved in gluconeogenesis are known to be PPAR*α* targets in nonruminants [182]. One of the three known promoter regions of bovine pyruvate carboxylase (*PC*), a key enzyme in gluconeogenesis, was activated by Wy-14643 when transfected as a construct with firefly luciferase into rat hepatoma cells, indicating a potential control of expression of this enzyme by PPAR*α* in ruminants [93]. However, the *PC* expression was not induced in MDBK cells treated with Wy-14643 or single LCFA [28]. The expression of *PC* was instead induced by cocktails of LCFA and particularly the concentration mimicking NEFA composition in dairy cows around parturition [183]. Therefore, an increase in gluconeogenesis via the activation of PPAR*α* in ruminants still needs to be fully proven.

It has been demonstrated that the high-glucose-induced downregulation of the glucose transport system in bovine endothelial cells is mediated by PPAR*β*/*δ* [68]. It was shown that activation of PPAR*β*/*δ* inhibits the expression of the solute carrier family 2 member 1 (or facilitated glucose transporter GLUT1) coupled with an increase in expression of calreticulin, a protein that increases degradation of GLUT1 mRNA. The condition tested in the study (i.e., high glucose) has probably little implication for ruminants, considering the low level of circulating glucose compared with nonruminants (<4 mM in dairy cows [176] versus ca. 5 mM in human and >6 mM in mouse [184]). However, the control of glucose transport by PPAR*β*/*δ* could have implications in milk synthesis, considering that GLUT1 is one of the most important glucose transporter and its expression increases drastically during lactation in mammary tissue of dairy cows [185]. Thus, this PPAR isotype could play a pivotal role in provision of glucose for lactose synthesis. Interestingly, in mammary gland during lactation, *PPARD* is significantly downregulated [186] concomitant with an increase in expression of several glucose transporters, including GLUT1 [70, 185]. If the suggested link is real, this offers the opportunity of using PPAR*β*/*δ* antagonists in order to improve milk production.

More recently, it was demonstrated that *PPARD* transcript in rumen epithelium of neonatal dairy calves is substantially more abundant than *PPARA* (see also Figure 1(b)), and its expression increased markedly from the milk-fed stage to the roughage-fed (i.e., high-structural fiber) stage at ~10 weeks of age [64]. The increase correlated with greater mass of the rumen, which suggested a potential link between PPAR*β*/*δ* and mechanisms driving ruminal epithelial cell development and proliferation [64].

## 10. What Controls Abundance of PPAR in Tissues?

The sensitivity of various tissues to PPAR isotype-specific agonists is closely related with the abundance of the specific isotype and other essential factors such as the abundance of coactivators or corepressors, LCFA, and hormones [76, 187–189]. As for nonruminants, the abundance of various isotypes in tissues appears to be directly related with the specific function they perform; for example, PPAR*γ* abundance is relatively high in lipogenic tissues while PPAR*α* is relatively high in tissues with elevated FA catabolic capacity (see Figure 1(a)). Besides tissue-specific distribution, other factors can control the abundance of PPAR isotypes in tissues.

Among factors controlling PPAR isotypes expression in ruminants (Supplementary Table 2), it is evident that several lipid molecules, some nutritionally relevant such as LCFA and retinoids, and propionate (likely indirectly via glucose and insulin) can affect expression of PPAR isotypes, with a different sensitivity based on tissue type. The expression of ruminant PPAR isotypes is also affected by physiological status, level of energy in the diet, mechanical cues (e.g., laminar flow, mechanical load), oxygen and peroxide levels, hormones, and other growth factors (Supplementary Table 2). In addition, data from several groups also suggest that the activation of PPAR*γ* increases expression of its own gene and, in the case of sheep, also the expression of *PPARA* (Supplementary Table 2). Interestingly, in bovine mammary epithelial cells several PPAR*γ* agonists decreased the expression of *PPARD*, with one case (ciglitazone) in which *PPARG* also was downregulated [69].

Overall the data presented in Supplementary Table 2 suggest that it is possible to increase or decrease the abundance, hence the sensitivity, of PPAR isotypes in ruminant tissues. Among the factors affecting the PPAR isotype expression, the more interesting from a nutrigenomics point of view are the LCFA and the level of dietary energy because they can be easily manipulated.

## 11. PPAR Isotype Activation during the Peripartal Period in Dairy Cattle: A Hypothesis

### 11.1. The Peripartal Condition

The transition from pregnancy into lactation (also called simply “transition period”) is one of the most stressful stages of the life of dairy cattle [190]. Physiologically, the transition period is a complex phenomenon intertwining various metabolic activities (e.g., lipid, glucose, protein) and functions (e.g., inflammatory response) of several organs and tissues (e.g., adipose tissue, mammary, liver, uterus, and immune system) [152, 190]. A key feature of the transition period from a metabolic and health standpoint is the increase in plasma of NEFA and ketone bodies (KB), both of which can be toxic above certain thresholds, and by a general decrease in both insulin sensitivity (except for the mammary gland) and blood insulin concentration [191]. The transition period is also characterized by inflammatory-like conditions as consequence of the release of proinflammatory cytokines, which along with NEFA affects directly liver functionality leading to poor performance [192].

The metabolic load placed on the liver of periparturient cows is exacerbated by this inflammatory-like conditions and also by the decrease in feed intake and the ensuing NEB, which often occurs as early as 10 days prior to parturition (reviewed in [193]). All of the above increase the risk of dairy cattle for developing metabolic disorders such as fatty liver [194] and ketosis [195, 196], but more importantly these disorders are tightly connected with other typical peripartal diseases [197]. Therefore, a smooth transition period is an important target in order to optimize performance and overall welfare of dairy cows. Interestingly, most of the above-described conditions (e.g., high NEFA, insulin insensitivity, fatty liver, inflammatory-like conditions) with the exception of the NEB are common to the metabolic syndrome that afflicts human [198].

### 11.2. PPAR Isotype Activation to Help Transition Dairy Cattle

It has been proposed previously that the PPAR isotypes are ideal targets for the prevention and cure of the metabolic syndrome in humans [199]. The use of PPAR*γ* agonists is a clinical approach currently in practice to treat insulin resistance, one of the main problems related with the metabolic syndrome [22, 200]. Similarly, it was proposed earlier that PPAR isotypes play a pivotal role in the physiological adaptation of dairy cattle to the transition period [36, 201]. It was proposed that fine-tuning the activity of PPAR*α* and PPAR*γ*, in particular, by nutritional approaches at specific time/s during the transition period might be a way to prevent and/or help the cows overcome metabolic disorders. Among nutritional approaches in order to affect PPARs, the saturated LCFA appear to be the most promising based on *in vitro* data (see above and [28]). The effects of saturated LCFA on PPARs activation and the consequent improvement of lipid metabolism appear to be supported by recent *in vivo* data [202]. In that study it was observed that the adaptations in lipid metabolism in dairy cows fed high-saturated fat compared with a low-fat control diet or a high-linseed diet (high in unsaturated LCFA) for up to 5 weeks pre-partum was better.

**Figure 4:**
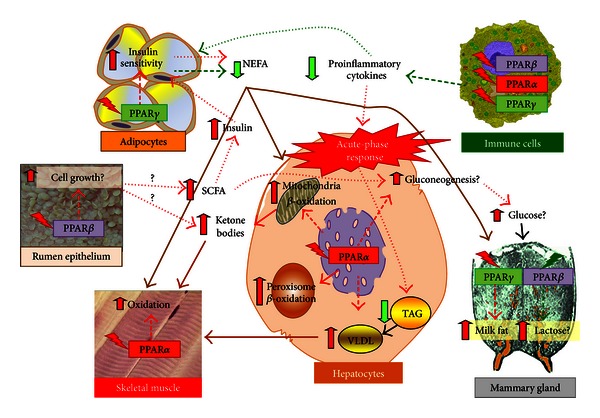
Improving transition from pregnancy into lactation in high producing dairy cows by nutrigenomics approach through PPAR isotypes: a hypothesis. The liver buffer cells from the excessive concentration of circulating nonesterified fatty acids (NEFA) by both catabolizing long-chain fatty acids (LCFA) with production of ketone bodies (KB) and esterifying them as triacylglycerol (TAG). The TAG are then accumulated in lipid droplets and packed into VLDL for release into the bloodstream. The liver is also induced by proinflammatory cytokines to produce positive acute phase proteins (+APP) taking away hepatic resources for normal liver functions. Despite the decrease in peripheral insulin concentration postpartum, the activation of PPAR*γ* prior to parturition can decrease NEFA postpartum through greater insulin sensitivity primarily on the adipose tissue. The activation of PPAR*β*/*δ* (PPAR*β* in the figure) via LCFA can increase rumen epithelium growth with consequent larger production of short-chain fatty acids (SCFA) including propionate, which stimulate insulin production, and butyrate, augmenting the KB in blood. The increased activation of PPAR*α* just before parturition and during the first 14 days postpartum in the liver and muscle can increase NEFA oxidation with greater proportion of KB produced per amount of NEFA uptake. The activation of PPAR*α* in the liver has the potential to increase gluconeogenesis and VLDL synthesis. The KB can serve as fuels by skeletal muscle instead of NEFA and glucose; both molecules are substrates for mammary gland. In this tissue, the activation of PPAR*γ* postpartum should increase or maintain milk fat. In addition, the inhibition of PPAR*β*/*δ* postpartum can potentially increase glucose import with a consequent increase in lactose synthesis, and hence, milk yield. The activation of PPAR isotypes just prior to parturition and during the first two weeks post-partum should diminish the inflammatory-like conditions preventing, on one hand, the stimulation of NEFA release and, on the other hand, hepatic acute-phase reaction, both determined by proinflammatory cytokines. This coordinated set of reactions should provide an ideal metabolic situation leading to a smoother transition from pregnancy into lactation, that is, allow the liver to allocate its resources for “normal” functions. As a consequence of this, the incidence of diseases typical of the peripartal period would be reduced, and hence, cows with higher performance and more healthy. Regular dashed arrows represent “effect on” due to PPAR isotype activation/inhibition, and round dot arrows denote secondary (or indirect) effects of PPAR isotype activation. In both cases red = activation or increase and green = inhibition or decrease.

In Figure 4 a qualitative hypothetical model describing the potential role of PPAR isotypes in transition dairy cows is depicted. That model rests on the well-established fact that the liver, adipose, rumen, skeletal muscle, immune system, and mammary gland play a crucial role in the adaptations leading to the onset of lactation. Other organs such as uterus, kidney, and pancreas also are crucial in this context but less is known about their molecular adaptations to lactation. In particular, data partly reviewed above strongly support a pivotal role of PPAR isotypes in the regulation of fertility and pregnancy; however, the overall effect of PPAR isotypes activation on fertility is not fully clear. In addition, the PPAR isotypes likely play a more important role before pregnancy compared with early lactation, when the cows are not yet cycling. Once the role of PPAR isotypes is better defined for the reproductive organs, it can become an important component of the overall model proposed.

Dairy cattle during the transition from pregnancy into lactation experience a multitiered set of adaptations aimed at allowing the mammary gland to begin and maintain lactogenesis. From a physiological perspective, the inherently low capacity of animals to consume enough dietary energy and the detrimental inflammatory-like conditions due to release of proinflammatory cytokines lead to the marked release of LCFA into the bloodstream from the adipose tissue. Those LCFA are mostly metabolized by the liver. A greater level of dietary energy in the form of nonstructural carbohydrate provided to the animal early postpartum can partly alleviate the negative shortfall in energy status; such approach would enhance production of short-chain fatty acids (SCFA), of which propionate metabolism via gluconeogenesis could serve as a trigger for greater insulin secretion [193]. The latter has been shown to promote rumen epithelial cell proliferation and might work in concert with PPARs to coordinate metabolism and development of these cells [64]. During the peripartal period, proinflammatory cytokines are released and induce the liver to produce +APP [176], taking away hepatic resources for normal liver functions (e.g., glucose synthesis, lipid metabolism, and ureagenesis) [175, 176, 197]. This condition effectively exacerbates the tissue's capacity to coordinate appropriately metabolism of lipid and to provide the required glucose to mammary gland for milk synthesis. The marked NEFA concentration is only partly oxidized by liver with the rest accumulating as TAG. The TAG are then packed into VLDL for release into the bloodstream, but at a lower rate relative to monogastrics [203]. An excessive accumulation of TAG can have detrimental effects on liver function [194].

We propose that the increased abundance pre-partum of PPAR isotypes and the timely and isotype-specific activation pre- or post-partum might be beneficial in preparing and allowing the animal to face the above-described conditions favoring a smooth transition into lactation. In particular the following:

- the greater abundance and activation of PPAR*γ* prepartum in adipose tissue can prevent the large NEFA surge due partly to an increase in insulin sensitivity, leading to reduced lipid overload on the liver with a consequent reduction of fatty liver, ketone body production, and any potential satiety effects (as consequence of high FA oxidation) [204]. The activation of PPAR*γ* postpartum in mammary gland can allow to increase or to maintain the amount of milk fat in the early stages of lactation when NEFA provide exogenous LCFA for the mammary gland. Several pieces of evidence support such expected effects in adipose tissue [82, 109] and mammary tissue [26, 33, 186];
- the greater abundance and activation of PPAR*α* in liver and skeletal muscle in postpartum relative to prepartum can increase oxidation of NEFA leading to a lower accumulation of lipid in the liver. The greater oxidation capacity of liver (i.e., increase ketone body synthesis per unit of NEFA oxidized) would help prevent any substantial alteration in the production of ketone bodies due to the systemic decrease of NEFA as a consequence of PPAR*γ* activation. The activation of PPAR*α* in liver might also increase gluconeogenesis rate, an essential process in ruminants particularly for milk synthesis. Another expected response would be increased VLDL synthesis and secretion by preventing the negative effect of the acute-phase reaction as a consequence of inflammatory-like conditions on apolipoproteins and other molecules involved in VLDL synthesis and TAG export. This suggestion is based on several pieces of evidence such as the observed negative association between apolipoprotein B100 or other VLDL components with inflammatory-like conditions in ruminants [170, 175, 176, 194, 205, 206];
- the activation of PPAR*α*, PPAR*γ*, and particularly PPAR*β*/*δ* in immune (e.g., neutrophils, macrophages) and endothelial cells might contribute to a reduction of the NEFA surge induced by proinflammatory cytokines [207] and also increase insulin sensitivity and prevent the negative effect of acute phase reaction on liver functionality [166, 176].

The hypothetical model for fine-tuning PPAR isotypes for prevention of metabolic disorders in transition dairy cows we propose (Figure 4) is indirectly supported by several *in vivo* and *in vitro* studies, but a number of major details remain to be understood. One of the most important pertains to the effects of LCFA on PPAR isotype activation and, particularly, on how they could be used to target activation of a particular PPAR isotype at a particular stage of the transition period. More detailed and mechanistic studies with LCFA, for example, effective dose/s of individual LCFA or mixtures, are essential in order for these nutrients to have practical application as proposed in our model (Figure 4).

## 12. Conclusions and Perspectives

The understanding of physiological roles of PPAR isotypes in ruminants has advanced incrementally over the last decade. There is enough direct and indirect evidence compiled to conclude that these NR are biologically relevant in this species. The data suggest that the harmonized activity of PPAR isotypes across tissues is one facet of the multitiered set of control points that evolved to coordinate metabolism and physiological responses to endogenous and exogenous ligands. The transition from pregnancy to lactation provides the clearest example of the need for control points to ensure the nourishment of the neonate offspring, while ensuring the fitness of the dam. At a fundamental level, the functional activity of PPARs during this physiological state provides an elegant example of the multitiered concept because it links biological molecules with cellular responses that encompass several tissues. The model proposed based on the most-current knowledge is quite complex and its full evaluation obviously requires an integrative systems approach, that is, several tissues at various levels (e.g., cells and the underlying molecular networks) need to be studied simultaneously considering their dynamic adaptation.

If the model/hypotheses proposed hold, it would become the first “true” nutrigenomics application in dairy cattle biosciences. Benefits would go beyond simply establishing the physiological role of PPAR isotypes in ruminants. Improving the transition from pregnancy into lactation means to provide benefits for farmers, dairy cattle, and the society as a whole. We envisage farmers modulating the LCFA in the diets of dairy cattle to fine-tune metabolism through PPAR isotypes. Today, the continuous development of high-throughput technologies and bioinformatics tools permits the study of complex phenomena as is the case of the transition from pregnancy to lactation in dairy cattle [152]. This is an exciting era for expanding scientific knowledge and, apparently, the proper one for nutrigenomics.

## Supplementary Material

Supplementary Table 1: Summary of the studies where activation of PPAR isotypes was performed in ruminants using synthetic agonists or other known natural ligands (e.g., 15-deoxy-Δ^12,14^-prostaglandin J2 for PPAR**γ**). The studies are sorted by year of publication.
Supplementary Table 2: Factors affecting the expression of PPAR isotype genes in various ruminant tissues/cells.

## Figures and Tables

**Table 1:** Activation of PPAR isotypes in ruminants by main long-chain fatty acids or glucose.

LCFA/glucose	Effect on PPAR isotype^@^			Method^#^	References
	PPAR*α*	PPAR*γ*	PPAR*β*/*δ*		
16:0	+++	+++	n/a	Indirect	[26, 28, 61]
18:0	+++	+++	n/a	Indirect	[26, 28]
c9-18:1	++	+	n/a	Indirect/Luciferase	[26, 28, 62]
t10-18:1	n/a^&^	+	n/a	Indirect	[26]
18:2	+	n/a	n/a	Indirect	[26, 28]
c9,t11-18:2	+	n/a	n/a	Indirect	[26, 28]
t10,c12-18:2	+	±	n/a	Indirect	[26, 28]
CLAmix^$^	+	n/a	n/a	Indirect	[36, 77]
20:0	++	n/a	n/a	Indirect	[26, 28]
20:4n-6	++	n/a	++*	Indirect/Luciferase	[68, 77]
20:5n-3	++	++	n/a	Indirect	[26, 28]
22:6n-3	+	n/a	n/a	Indirect	[26, 28]
Glucose	No	n/a	++	Luciferase	[68]

^@^+++: strong agonist; ++: agonist; +: weak agonist; ±: mixture between agonist and antagonist.

*The 12-HETE, a metabolite of the 20:4n-6 is the actual agonist.

^#^Indirect: the effect on PPAR isotype target genes was uncovered by the use of specific PPAR synthetic agonists; luciferase: the use of the PPRE-luciferase construct to test activation of PPAR by agonists.

^$^A mixture (ca. 50% each) of the t10,c12- and c9,t11-conjugated 18:2

^&^Not available.

**Table 2:** PPAR isotype target genes in ruminants grouped by main biological function.

Gene	HUGO gene name	Tissue/cells^1^	PPAR^2^	Reference
Fatty acid import and activation				

*ACSL1 *	Acyl-CoA synthetase long-chain family member 1	MDBK	*⇑* PPAR*α*	[28, 61]
		Liver	*⇑* PPAR*α*	[78]
*ACSL3 *	Acyl-CoA synthetase long-chain family member 3	MDBK	*⇑* PPAR*α*	[28]
*CD36 *	Thrombospondin receptor	MDBK	*⇑* PPAR*α*	[28]
		BAEC	*⇑* PPAR*γ*	[79]
		MAC-T	*⇔* PPAR*γ*	[26]
*FABP4 *	Fatty acid binding protein 4	Muscle	*⇑* PPAR*γ*	[80]^#^
		MDBK	*⇑* PPAR*α*	[28]
		MAC-T	*⇔* PPAR*γ*	[26]
*LPL *	Lipoprotein lipase	sP. adipose	*⇑* PPAR*γ*	[81]
		MAC-T	*⇔* PPAR*γ*	[26]
		bS. adipose	*⇔* PPAR*γ*	[82]

Fatty acid synthesis				

*ACACA *	Acetyl-CoA carboxylase alpha	MAC-T	*⇑* PPAR*γ*	[26]
*FASN *	Fatty acid synthase	MAC-T	*⇑* PPAR*γ*	[26, 83]
		bS. adipose	*⇔* PPAR*γ*	[82]
		bS. adipose	*⇓* PPAR*γ*	[84]
*INSIG1 *	Insulin induced gene 1	MAC-T	*⇑* PPAR*γ*	[26]
*SCD *	Stearoyl-CoA desaturase (delta-9-desaturase)	MDBK	*⇑* PPAR*α*	[28]
*SREBF1 *	Sterol regulatory element binding factor 1	MAC-T	*⇑* PPAR*γ*	[26]
		MDBK	*⇑* PPAR*α*	[28]

Fatty acid oxidation				

*ACADVL *	Acyl-CoA dehydrogenase, very long chain	MDBK	*⇑* PPAR*α*	[61]
		Liver	*⇑* PPAR*α*	[78]
*ACOX1 *	Acyl-coenzyme A oxidase 1	MDBK	*⇔* PPAR*α*	[61]
		Liver**	*⇑* PPAR*α*	[78]
*CPT1A *	Carnitine palmitoyltransferase 1A (liver)	MDBK	*⇑* PPAR*α*	[28, 36, 61]
		Liver**	*⇑* PPAR*α*	[78]
*CPT2 *	Carnitine palmitoyltransferase 2	PAEC	*⇑* PPAR*γ*	[85]
*CRAT *	Carnitine O-acetyltransferase	PAEC	*⇑* PPAR*γ*	[85]
*CYP4A11 *	Cytochrome P450, family 4, subfam. A, polypeptide 11	Liver	*⇑* PPAR*α*	[78]

Triacylglycerol synthesis				

*AGPAT6 *	1-acylglycerol-3-phosphate O-acyltransferase 6	MAC-T	*⇑* PPAR*γ*	[26]
*DGAT1 *	Diacylglycerol O-acyltransferase 1	MAC-T	*⇑* PPAR*γ*	[26]
*LPIN1 *	Lipin 1	MAC-T	*⇑* PPAR*γ*	[26]
		MDBK	*⇑* PPAR*α*	[28, 61]
*LPIN3 *	Lipin 3	MDBK	*⇑* PPAR*α*	[28, 78]

Cholesterol synthesis				

*HMGCR *	3-Hydroxy-3-methylglutaryl-CoA reductase	MDBK	*⇑* PPAR*α*	[28]
*SREBF2 *	Sterol regulatory element binding transcription factor 2	MAC-T	*⇑* PPAR*γ*	[26]

Signaling molecules				

*ANGPTL4 *	Angiopoietin-like 4	Liver	*⇑* PPAR*α*	[56, 86]^++^
		MDBK	*⇑* PPAR*α*	[28]
*FGF21 *	Fibroblast growth factor 21	Liver	*⇑* PPAR*α*	[86]^++^
*EDN1 *	Endothelin 1	BAEC	*⇓* PPAR*α*	[87]
			*⇓* PPAR*γ*	
*LEP *	Leptin	bS. adipose	*⇑* PPAR*γ*	[84]
*NOS3 *	Nitric oxide synthase 3 (endothelial cell)	BAEC	*⇑* PPAR*α*	[88]
*PTGS2 *	Prostaglandin-endoperoxide synthase 2	BEND	*⇑* PPAR*α*	[44]
			*⇑* PPAR*γ*	[44]
		pBESC	*⇑* PPAR*α*	[77]
		MAC-T	*⇔* PPAR*γ*	[26]
*SPP1 *	Osteopontin	MDBK	*⇑* PPAR*α*	[28]
*VEGF *	Vascular endothelial growth factor	BAEC	*⇑* PPAR*γ*	[89]

Other functions				

*CDKN2A *	Cyclin-dependent kinase inhibitor 2A	BAEC	*⇓* PPAR*γ*	[90]
*GAPDH* ^ $^	Glyceraldehyde-3-phosphate-dehydrogenase	s. ASC	*⇑* PPAR*γ*	[91]
			*⇑* PPAR*β*/*δ*	
*OLR1 *	Oxidized low density lipoprotein receptor 1	BAEC	*⇑* PPAR*α*	[92]
			*⇓* PPAR*γ*	[46]
*PC *	Pyruvate carboxylase	Hepatoma*	*⇑* PPAR*α*	[93]
		MDBK	*⇔* PPAR*α*	[28]
*SLC2A1 *	Solute carrier family 2, member 1	BAEC	*⇓* PPAR*β*/*δ*	[68]
*TERF2 *	Telomeric repeat binding factor 2	BAEC	*⇑* PPAR*γ*	[90]

PPAR activation-related functions				

*PPARA *	Peroxisome-proliferator-activated receptor alpha	BAEC	*⇑* PPAR*α*	[62]
		MDBK	*⇔* PPAR*α*	[28, 61]
		Liver	*⇑* PPAR*γ*	[81]
		Muscle**	*⇑* PPAR*γ*	[81]
*PPARG *	Peroxisome-proliferator-activated receptor gamma	bEPC	*⇑* PPAR*γ*	[94]
		MAC-T	*⇔* PPAR*γ*	[26]
*PPARGC1A *	PPAR*γ*, coactivator 1 alpha	s. Muscle	*⇑* PPAR*γ*	[81]

^1^Acronyms: BAEC: Bovine Aortic Endothelial Cells; BEND: Bovine Endometrial Cells; bEPC: bovine renal Epithelial cells; BRCP: Bovine Retinal Capillary Pericytes; bS. Adipose: bovine subcutaneous adipose; pBESC: primary (16-day cycle) bovine endometrial stromal cells; MDBK: Madin-Darby Kidney Cell Line; PAEC: ovine pulmonary arterial endothelial cells; sP.adipose: sheep perirenal adipose; ^1^s. ASC: sheep adipose stem cells; s. Muscle: sheep muscle.

^2^The PPAR activated by the treatment with a different effect on expression of the target gene (*⇑* induction; *⇓* inhibition; *⇔* no change).

*Rat hepatoma was transfected with bovine PC promoter region.

**The increase in expression was with *P* < 0.10 but *P* > 0.05.

^$^The activity and not the mRNA expression of GAPDH was measured.

^#^Inferred based on the high correlation of expression between *PPARG* and *FABP4.*

^++^Inferred based on hepatic mRNA expression in studies with peripartal cows and undernutrition ketosis [56, 86, 95] (see main body of the paper for details).

## Abbreviations

+APP: Positive acute phase proteins
*ACOX1*: Acyl-coenzyme A oxidase 1, palmitoyl
AF-1: Activation Function 1 or ligand-independent activation function
*ANGPTL4*: Angiopoietin-like 4
bMEC: Bovine primary mammary epithelial cells
CARLA: Coactivator-dependent receptor ligand assay
*CCL2*: Chemokine (C-C motif) ligand 2
CLA: Conjugated linoleic acid
*CPT1A*: Carnitine palmitoyltransferase 1A (liver)
*CPT2*: Carnitine palmitoyltransferase 2
*CRAT*: Carnitine O-acetyltransferase
CXCL6: Chemokine (C-X-C motif) ligand 6
EPA: Eicosapentaenoic acid
FA: Fatty acid(s)
*FABP*: Fatty acid binding protein
*FGF21*: Fibroblast growth factor 21
*GAPDH*: Glyceraldehyde-3-phosphate dehydrogenase
GLUT1: Glucose transporter 1
IL-8: Interleukin 8
*INSIG1*: Insulin induced genes 1
*INSIG2*: Insulin induced genes 2
KB: Ketone bodies
LBD: Ligand binding domain
LCFA: Long-chain fatty acid(s)
LCFA-CoA: LCFA-coenzyme A (i.e., activated LCFA)
LDL: Low density lipoprotein(s)
*LPIN1*: LiPIN 1 (coding for a phosphatidate phosphatase)
*LPL*: Lipoprotein lipase
LPS: Lipopolysaccharide
*LXR*: Liver X receptor
MAC-T: Bovine mammary alveolar cells transfected with simian virus-40 (SV-40) large T-antigen
MDBK: Madin-Darby bovine kidney cells
MIQE: Minimum Information for Publication of Quantitative Real-Time PCR Experiments
NEB: Negative energy balance
NEFA: Nonesterified fatty acid(s)
*NFKB1*: Nuclear factor of kappa light polypeptide gene enhancer in B-cells 1
NR: Nuclear receptor(s)
*PC*: Pyruvate carboxylase
PG: Prostaglandin(s)
PGJ2: 15-Deoxy-delta-12,14-prostaglandin J2
PMN: Polymorphonuclear cells
PPAR: Peroxisome proliferator-activated receptor
PPRE: PPAR response element
PUFA: Polyunsaturated fatty acid(s)
qPCR: Quantitative Real-Time reverse transcription polymerase chain reaction
*RXR*: Retinol X receptor
*SCAP*: SREBP cleavage-activating protein
*SCD*: Stearoyl-CoA desaturase
SCFA: Short-chain fatty acid(s)
siRNA: Short interference RNA
*SREBF1*: Sterol regulatory element-binding transcription factor 1
TAG: Triacylglycerol(s)
TF: Transcription factor(s)
TNF: Tumor necrosis factor(s)
TZD: Thiazolidinedione
*VEGF*: Vascular endothelial growth factor
VLDL: Very low density lipoprotein(s)
Wy-14643: Pirinixic acid.
