# Supplementary material for: Functional Role of PPARs in Ruminants: Potential Targets for Fine-Tuning Metabolism during Growth and Lactation
Source: PPAR Res. 2013 Apr 29;2013:684159. doi: 10.1155/2013/684159 (PMC3657398; doi:10.1155/2013/684159)
Supplement: Supplementary file 1 — Supplementary Table 1: Summary of the studies where activation of PPAR isotypes was performed in ruminants using synthetic agonists or other known natural ligands (e.g., 15-deoxy-Δ12,14-prostaglandin J2 for PPARγ). The studies are sorted by year of publication. Supplementary Table 2: Factors affecting the expression of PPAR isotype genes in various ruminant tissues/cells. [file 684159.f1.docx]

**SUPPLEMENTARY TABLES.**

Bionaz et al., Functional Role of PPAR in Ruminants: Potential Targets for Fine-Tuning Metabolism during Growth and Lactation. PPAR Research. 2013

S. TABLE 1. Summary of the studies where activation of PPAR isotypes was performed in ruminants using synthetic agonists or other known natural ligands (e.g., 15-deoxy-Δ^12,14^-prostaglandin J2 for PPARγ). The studies are sorted by year of publication.

| Species, n^1^  Stage/tissue^2^ | Type year | Agonist | Target | Dose | Way | Time treatm. | Main effects^2^ | Authors’ conclusions | Ref. |
| --- | --- | --- | --- | --- | --- | --- | --- | --- | --- |
| Bovine  3  Lutein cells  Primary | *In vitro*  1998 | PGJ2  TZD | PPARγ  PPARγ | 100 nM  1 μM | Media | 24 h | ⇑ Progesterone  ⇑ Progesterone | PPARγ controls progesterone synthesis | [[1](#_ENREF_1)] |
| Bovine  3x3  SVC  Primary | *In vitro*  1998 | TZD | PPARγ | 25 μM | Media | 10-14d | ⇑ adipogenesis | PPARγ controls adipogenesis | [[2](#_ENREF_2)] |
| Bovine  3x3  BAEC  Primary | *In vitro*  1999 | PGJ2 | PPARγ | 10 μM | Media | 24 h | ⇑ activation PPRE_3_-luciferase | BAEC cells contain responsive PPARγ | [[3](#_ENREF_3)] |
| Sheep  4-14  ASC  Primary | *In vitro*  1999 | Wy-14643  Rosiglitazone  Bezafibrate | PPARα  PPARγ  PPARβ/δ | 100 μM  100 nM  100 μM | Media | 10d | ⇑ activity of GAPDH | PPARγ controls adipogenesis | [[4](#_ENREF_4)] |
| Bovine  5  BMEC-b  Not primary | *In vitro*  1999 | PGJ2 | PPARγ | 10 μM | Media | 20 h | ⇓ cell viability | PGJ2 induces endothelial apoptosis | [[5](#_ENREF_5)] |
| Bovine  3  BAEC  Not primary | *In vitro*  1999 | Fenofibriate  Wy-14643  Troglitazone  BRL49653 | PPARα  PPARα  PPARγ  PPARγ | 100 μM  1 μM  1 μM  10 nM | Media | 24 h | ⇓ ET-1 production  ⇓ ET-1 production  ⇓ ET-1 production  ⇓ ET-1 production | PPAR activators repress thrombin-induced ET-1 secretion in endothelial cells | [[6](#_ENREF_6)] |
| Bovine  3  BAEC  P = 3 to 5 | *In vitro*  2001 | Wy-14643  Fenofibrate  PGJ2  Troglitazone | PPARα  PPARα  PPARγ  PPARγ | 250 μM  100 μM  3 μM  15 μM | Media | 16 h | ⇑ LOX-1  ⇑ LOX-1  ⬄ LOX-1  ⬄ LOX-1 | Endothelial LOX-1 can be upregulated by PPARα | [[7](#_ENREF_7)] |
| Bovine  3  BAEC  P = 12 | *In vitro*  2001 | Pioglitazone  Troglitazone  Fenofibrate  Wy-14643  PGJ2 | PPARγ  PPARγ  PPARα  PPARα  PPARγ | 10 μM  10 μM  200 μM  200 μM  10 μM | Media | 24 h | ⇓*OLR1* after ⇑ by TNFα  ⇓*OLR1* after ⇑ by TNFα  ⬄*OLR1* after ⇑ by TNFα  ⬄*OLR1* after ⇑ by TNFα  ⇓*OLR1* after ⇑ by TNFα | PPARγ activators inhibit TNFα-induced  LOX-1 expression | [[8](#_ENREF_8)] |
| Bovine  4-6  BAEC  P = ? | *In vitro*  2001 | Pioglitazone  Troglitazone | PPARγ  PPARγ | 10 nM  10 nM | Media | 5 days | Both treatments:  ⇑ DNA synth. And proliferat.  ⇑ CNP secretion  ⇓ endothelin | TZD modulates  endothelial functions (growth and vasoactive substances) | [[9](#_ENREF_9)] |
| Bovine  6 (3)  Steers treated with rbTNF | *In vivo*  2001 | 2,4-TZD | PPARγ | 2 mg/kg/day | Subcut. injection | 9 d | ⇓ NEFA, ⇓ glucose  ⇓ insulin, ⇓ glucagon  ⇑ glucose nAUC  ⇓ insulin nAUC | 2,4-TZD counteracts insulin-resistance with potential beneficial effects | [[10](#_ENREF_10)] |
| Bovine  4 exp x 3 rep  BAEC  P = 6 | *In vitro*  2002 | Ciprofibrate | PPARα | 50 μM | Media | 6 h | ⇓ endothelin 1 (only with added CsA) | PPARα prevents negative effects of CsA. Mevalonate interferes with PPARα | [[11](#_ENREF_11)] |
| Goat  6 (3)  Lactating  liver | *In vivo*  2002 | Wy-14643 | PPARα | 40 mg/kg/day | Oral | 14 days | ⇓ blood cholesterol  ⇑ hepatic β-oxidation  ⇑ hepatic aromatase | Goat weak responder to PPAR agonist | [[12](#_ENREF_12)] |
| Sheep  4-7  Granulosa  Pituitary  Primary | *In vitro*  2003 | Rosiglitazone | PPARγ | 10 μM | Media | 24-96h | ⇓ cell proliferation  ⇑ progesterone secretion  ⇔ LH secretion  ⇔ FSH secretion | PPARγ affects  follicular maturation and corpus luteum functionality | [[13](#_ENREF_13)] |
| Bovine  3  BAEC  P = 3 to 5 | *In vitro*  2004 | Fenofibrate  Bezafibrate  Wy-14643  Rosiglitazone | PPARα  panPPAR  PPARα  PPARγ | 50 μM  50 μM  50 μM  50 μM | Media | 48 h | ⇑ eNOS  ⇑ eNOS  ⇑ eNOS  ⬄ eNOS | The data might explain the PPARα vasodilator function | [[14](#_ENREF_14)] |
| Bovine  5  BAEC  Primary | *In vitro*  2004 | Fenofibrate  Wy-14643  Troglitazone  PGJ2 | PPARα  PPARα  PPARγ  PPARγ | 100 μM  250 μM  15 μM  3 μM | Media | 16 h  3-48 h  16 h  16 h | ⇑ LOX-1  ⇑ LOX-1  ⇔ LOX-1  ⇔ LOX-1 | Endothelial LOX-1 expression is  upregulated by PPARα | [[15](#_ENREF_15)] |
| Bovine  5-7  BAEC  P = 5-9 | *In vitro*  2004 | Troglitazone | PPARγ | 20 μM | Media | 12 h | ⇑ endothelial nitric oxide  ⬄ eNOS  ⇑*VEGF*, ⇑ KDR-Flk-1  ⇑ Akt phosphorylation  ⇑ phosp. Ser^1179^ eNOS  ⇑ dephosp. Ser^116^ eNOS | PPARγ activation increases endothelial nitric oxide | [[16](#_ENREF_16)] |
| Bovine  4  BRCP  P = 2-4 | *In vitro*  2005 | Troglitazone | PPARγ | 20 μM | Media | 24 h | ⇑ nitric oxide  ⇑*NOS2* | Troglitazone restores (induces) the production of nitric oxide | [[17](#_ENREF_17)] |
| Bovine  4-6  BAEC  N/A | *In vitro*  2006 | Troglitazone | PPARγ | 20 μM | Media | 20 min | ⇓ protein synthesis  ⇓ p70S6K activity  ⇑ p70S6K- PP2A assoc.  Not effect when PPARγ was inhibited | Troglitazone decreases protein synthesis independent of PPARγ | [[18](#_ENREF_18)] |
| Bovine  N/A  BAEC  P < 4 | *In vitro*  2006 | Bezafibrate | panPPAR | 50-200 μM | Media | 2 h | ⇑*NOS3*, eNOS.  ⇑ nitric oxide  ⇑ phosph. eNOS-ser^1179^  ⇑ *NOS3* half-life  ⇑ MAPK phosphoryl. | Anti-atherosclerotic and anti-hypertension benefits of bezafibrate | [[19](#_ENREF_19)] |
| Bovine  2 exp.× 2 duplicates  BEND | *In vitro*  2006 | Wy-14643  Ciglitizone  Carbacyclin^&^ | PPARα  PPARγ  PPARδ | 10 μM  10 μM  1 μM | Media | 24 h | ⇑ PGF2α, PGE2, *PTGS2*  ⇑*PTGS2*  ⇑ PGF2α*, PGE2 | PPARα and δ are expressed in BEND and involved in regulation of PG synthesis | [[20](#_ENREF_20)] |
| Bovine  3-8  pBESC  primary | *In vitro*  2007 | Wy-14643  Ciprofibrate | PPARα  PPARα | 50 μM  50 μM | Media | 6, 24h | ⇑*PTGS2*  ⇑*PTGS2* | PPARα is central in controlling *PTGS2* expression | [[21](#_ENREF_21)] |
| Bovine  4  bEPC  P = 10-20 | *In vitro*  2007 | Rosiglitazone | PPARγ | 25 μM | Media | 6, 24 h | ⇑*PPARG* and PPARγ.  ⇑nuclear PPARγ binding | PPARγ may be important for recovery after oxidative stress | [[22](#_ENREF_22) ] |
| Bovine  14 (7)  Peripartum | *In vivo*  2007 | 2,4-TZD | PPARγ | 4 mg/kg/day | Jugular infusion | 25 d prepartum | *Prepartum:*  ⇓NEFA,⇓BHBA,⇑DMI*  *Postparum:*  ⇑ insulin; ⇑DMI* | TZD can improve metabolism of peripartum cows | [[23](#_ENREF_23)] |
| Bovine  3  MDBK  P >230 | *In vitro*  2008 | Wy-14643  Rosiglitazone | PPARα  PPARγ | 50 μM  10 μM | Media | 24 h | ⇑*CPT1A*  ⇑*LPL^#^*  ⬄*ACOX1* | MDBK cells are responsive to PPAR agonists | [[24](#_ENREF_24)] |
| Bovine^@^  2  bMEC  primary | *In vitro*  2008 | PGJ2  MC-555  Ciglitazone  Rosiglitazone  Troglitazone | PPARγ | 10 μM | Media | 48 h | ⇑*IL8*,*CXCL6*,*FTH*  ⇓*IL1*,*IL6,*⇑*TNF*  ⇓*IL6*,*IL1*,*IL8* ⇑*TNF*  ⇓*IL6*,*IL1*,⇑*CCL2*,*TNF*  ⇓*IL6*,*IL8*,*CXCL6*,⇑*CCL2* | PPARγ ligands can alter inflammatory response | [[25](#_ENREF_25)] |
| Bovine  36 (12)  Peripartum | *In vivo*  2009 | 2,4-TZD | PPARγ | 0, 2, or 4 mg/kg/day | Jugular infusion | 21 d prepartum | *Prepartum:*  ⇑BHBA,⇑DMI  *Postparum:*  ⇓NEFA,⇑glucose  ⇓TAG, ⇑glycogen liver  ⇑DMI, ⇑BCS, ⇓ FC milk  ⇓first ovulation | TZD improves metabolic health and DMI and may decrease use of fat reserves | [[26](#_ENREF_26)] |
| Bovine  3  MDBK  P = 112 | *In vitro*  2009 | Wy-14643 | PPARα | 150 μM | Media | 24 h | ⇑*CPT1A,* ⇑*ACSL1*  ⇑*ACADVL*  ⬄*LPIN1,*⬄*ACOX1*  ⬄*PPARGC1A,*⬄*PPARA* ⬄SREBF1 | Maximal response to PPARα agonist at 18 h | [[27](#_ENREF_27)] |
| Bovine  3  MACT | *In vitro*  2009 | Rosiglitazone | PPARγ | 10 μM | Media | 12 h | ⇑*ACACA*, ⇑*FASN*  ⇑*LPIN1*, ⇑*AGPAT6*  ⇑*DGAT1*, ⇑*SREBF1*  ⇑*SREBF2*, ⇑*INSIG1* | PPARγ plays a role in regulation of milk fat synthesis | [[28](#_ENREF_28)] |
| Sheep  14 (7 ewes)  Adipose, liver, and muscle of late gest. fetuses | *In vivo*  2009 | Rosiglitazone | PPARγ | 4.28 mg/fetus/d | Intrafetal | 16 days | ⇓ insulin  ⇑*LPL* perirenal adipose.  ⇑*ADIPOQ* perir. adipose  ⇑*PPARA* liver  ⇑*PPARGC1A* muscle  ⇑*PPARA** muscle | Activation of PPARγ as potential programming of postnatal obesity | [[29](#_ENREF_29)] |
| Bovine  15 (5)  Weaned  Liver | *In vivo*  2010 | Clofibrate | PPARα | 62.5 mg/kg/day | Oral | 5 days | ⇓ intake;  ⇑ liver weight  ⇑ catabolism 16:0  ⇑*ACADVL*  ⇑*ACSL1*  ⇑*CYP4A11*  ⇑*CPT1A** and *ACOX1** | Bovine liver responds to PPAR agonist but to lower extent than rodents | [[30](#_ENREF_30)] |
| Bovine  4  BAEC  P = N/A | *In vitro*  2010 | WY14643  Troglitazone  GW501516 | PPARα  PPARα  PPARβ/δ | 60 μM  30 μM  0.1-1 μM | Media | 48 h | Only GW501516:  ⇓ rate of hexose transport  ⇓*SLC2A1* and GLUT1  ⇑*CALR* | Glucose autoregulates its up-take through PPARβ/δ | [[31](#_ENREF_31)] |
| Bovine  30  Adipocytes  From ASC | *In vitro*  2010 | Rosiglitazone | PPARγ | 20 μM | Media | ? | ⇑*PPARG* | Used as positive control during differentiation | [[32](#_ENREF_32)] |
| Bovine^%^ 3 Adipocytes from SVC | *In vitro* 2010 | RosiglitazoneTroglitazone | PPARγ PPARγ | 1 μM 5 μM | Media Media | 8 d | Both treatments: ⇑*PPARG*,*FABP4,ACACA* ⇑GAPDH | PPARγ agonists improve adipogenic differentiation | [[33](#_ENREF_33)] |
| Bovine  32 (8 +8)  Prepartum  Adipose | *In vivo*  2011 | 2,4-TZD | PPARγ | 4 mg/kg/day | Jugular infusion | 14 d prepartum | ⇑glucose, ⇑insulin  ⇑neg. glucose AUC*  No Δ gene expression | TZD improves metabolic health and food intake and may decrease use of fat reserves | [[34](#_ENREF_34)] |
| Bovine  40 (8 + 8)  Prepartum  Adipose | *In vivo*  2011 | 2,4-TZD | PPARγ | 0, 2, or 4 mg/kg/day | Jugular infusion | 21 d prepartum | *Prepartum:*  ⇑TNFα  *Postpartum:*  ⇑leptin*,⇑TNFα  ⇑*PPARG*, ⇓*FASN*  ⬄*LPL,* ⬄*LEP^$^* | TZD affects leptin, increases plasma TNFα and PPARγ expression in adipose tissue | [[35](#_ENREF_35)] |
| Bovine  3/treatment  BAEC  P = 3-6 | *In vitro*  2011 | Pioglitazone | PPARγ | 10 μM | Media | 3, 6, 12, 24 h | ⇑ telomerase activity  ⇑*TERF2* and TERF2  ⇓*CDKN2A*  ⇑ phosph. survival Akt | Pioglitazone might prevent age-related dysfunctions of vascular cells | [[36](#_ENREF_36)] |
| Bovine  3  PBMC  heifers | *In vitro*  2011 | Rosiglitazone | PPARγ | 10 μM | Media | 1h prior TNFα (tot 25h) | ⇓TNFα  ⇓NF-κBp65* | PPAR-γ agonists may attenuate the proinflammatory response induced by LPS | [[37](#_ENREF_37)] |
| Bovine  3  MDBK  P = 113 | *In vitro*  2012 | Wy-14643 | PPARα | 150 μM | Media | 6 h | ⇑*ACSL1*, *ACSL3*  ⇑*ANGPTL4, CD36*  ⇑*CPT1A, FABP4*  ⇑*HMGCR, LPIN1, LPIN3*  ⇑*SCD, SPP1, SREBF1* | Activation of PPARα potentially increases lipid metabolism | [[38](#_ENREF_38)] |
| Bovine  8-9/treatment  Liver, mucle, S.C.  Beef bulls | *In vivo*  2012 | 2,4-TZD | PPARγ | 8 mg/70 kg/day | Oral | 196 d | ⇓all *PPARs* in liver  ⇑*PPARA* in muscle | 2,4-TZD treatment improve overall lipid metabolism | [[39](#_ENREF_39)] |
| Ovine  4-8/treatment  PAEC  Primary | *In vivo*  *In vitro*  2012 | Rosiglitazone  siRNA | PPARγ | 3 mg/kg/ day | Oral | 4 weeks | ⬄ carnitine homeostasis^>^  ⇓*CPT2,CRAT* | PPAR-γ activation might prevent endothelial dysfunction | [[40](#_ENREF_40)] |

^1^total number of animal used (number of animal per treatment) or replicates/treatment if cell culture

^2^⇑ denotes induction/increase; ⇓ denotes inhibition/decrease; ⬄ denotes no change.

# The authors were never able to replicate the finding, likely due to the very low expression of LPL in those cells

* The results suggested tendency (P<0.10)

^$^ The 2 mg/kg/day treatment decreased *LEP* expression

^&^ Carbacyclin activates both PPARα and PPARβ/δ

^@^ Effect on PPAR isotype expression in this experiment is reported in S. Table 2

^%^ Effects of PPARγ agonists was additive to the adipogenic differentiation medium

^>^The treatment with rosiglitazone prevented the disruption of the carnitine homeostatis by avoiding the decrease in expression/translation of CPT1A, CPT2, and CRAT and the mitochondrial dysfunction associated with increased pulmonary blood flow.

**Abbreviations**:

*ACACA* = acetyl-CoA carboxylase alpha;

*ACADVL* = acyl-CoA dehydrogenase, very long chain;

*ACSL1* and *3* = acyl-CoA synthetase long-chain family member 1 and 3;

adip. = adipose tissue;

ASC = adipose stem cells;

*ADIPOQ* = adiponectin;

*AGPAT6* = 1-acylglycerol-3-phosphate O-acyltransferase 6 (lysophosphatidic acid acyltransferase, zeta);

Akt = serine/threonine protein kinase;

*ANGPTL4* = angiopoietin-like 4;

AUC = area under the curve after insulin challenge;

BAEC = bovine aortic endothelial cells;

BMEC-b = brain bovine microvascular endothelial cells;

bMEC = bovine mammary epithelial cells;

BCS = body condition score;

BEND = bovine endometrial cells;

bEPC = bovine renal epithelial cells;

BHBA = β-hydroxybutyrate;

BRCP = bovine retinal capillary pericytes;

*CALR* = calreticulin;

*CCL2* = chemokine (C-C motif) ligand 2;

*CD36* = thrombospondin receptor;

*CDKN2A* = cyclin-dependent kinase inhibitor 2A (p16 senescence marker);

*CPT1A* = carnitine palmitoyltransferase 1A (liver);

CNP = C-type natriuretic peptide;

CsA = cyclosporine A;

*CXCL6* = chemokine (C-X-C motif) ligand 6;

*DGAT1* = diacylglycerol O-acyltransferase 1;

DMI = dry matter intake;

eNOS = endothelial nitric oxide synthase;

ET-1 = endothelin 1;

*FABP4* = fatty acid binding protein 4;

*FASN* = fatty acid synthase;

*FTH* = ferritin heavy chain

FC milk = 3.5% fat-corrected milk;

GLUT1 = glucose transporter 1;

*HMGCR* = 3-hydroxy-3-methylglutaryl-CoA reductase;

*IL1B* = interlecukin 1β; *IL6* = interleukin 6;

*IL8* = interleukin 8;

*INSIG1* = insulin induced gene 1;

KDR-Flk-1 = kinase insert domain protein receptor or VEGF receptor 2;

LOX-1 = lectin-like oxidized LDL receptor;

*LPIN1* and *3* = lipin 1 and 3;

MDBK = Madin-Darby Bovine Kidney cells;

N/A = not available data;

nAUC = net area under the glucose response curve;

NEFA = non-esterified fatty acids;

*NOS2* = nitric oxide synthase 2, inducible;

*NOS3* = nitric oxide synthase 3, endothelial cell;

*OLR1* = oxidized low density lipoprotein (lectin-like) receptor;

1P = passage number of cells;

p70S6K = ribosomal protein S6 kinase, 70kDa;

PAEC = pulmonary arterial endothelial cell;

pBESC = primary (16 days cycle) bovine endometrial stromal cells;

panPPAR = able to activate all the three PPAR isotypes;

PBMC = peripheral blood mononuclear cells;

PGF2α = prostaglandin F2 alpha;

PGE2 = prostaglandin E2;

PGJ2 = 15-deoxy-12,14-prostaglandin J2;

PP2A = protein phosphatase A2;

*PPARGC1A* = peroxisome proliferator-activated receptor gamma, coactivator 1 alpha;

*PPARA*= peroxisome proliferator-activated receptor alpha;

*PPARD* = peroxisome proliferator-activated receptor beta/delta;

*PPARG* = peroxisome proliferator-activated receptor gamma;

*PTGS2* = prostaglandin-endoperoxide synthase 2;

S. C. = subcutaneous;

*SCD* = stearoyl-CoA desaturase (delta-9-desaturase);

*SLC2A1* = solute carrier family 2 (facilitated glucose transporter), member 1;

*SPP1* = secreted phosphoprotein 1 (osteopontin);

*SREBF1* and *2* = sterol regulatory element binding transcription factor 1 and 2;

SVC = Stromal-Vascular Cells from bovine adipose tissue;

TAG = triacylglycerol;

*TERF2* = telomeric repeat binding factor 2;

TNFα = tumor necrosis factor alpha;

TZD = thiazolidinedione;

Δ = variation;

S. TABLE 2. Factors affecting the expression of PPAR isotype genes in various ruminant tissues/cells.

| Effector | PPAR affected | Effect^1^ | Species | Tissue^2^ | Reference |
| --- | --- | --- | --- | --- | --- |
| *Fatty acids and other lipids* | | | | | |
| *Propionate* | PPARγ | **⇑** | Ovine | Subcutan. adipose | [[41](#_ENREF_41)] |
| *16:0* | PPARα | **⇑** | Bovine | MDBK* | [[27](#_ENREF_27)] |
|  |  | **⇔** |  | MDBK* | [[38](#_ENREF_38)] |
| *Trans18:1* | PPARα | **⇑** | Bovine | Liver | [[42](#_ENREF_42)] |
| *CLA* | PPARγ | **⇑** | Buffalo | Granulosa cells | [[43](#_ENREF_43)] |
|  |  | **⇔** | Bovine | Liver | [[44](#_ENREF_44)] |
|  |  | **⇔**  **⇔** |  | MAC-T  SVC | [[28](#_ENREF_28)]  [[33](#_ENREF_33)] |
|  | PPARα | **⇔** | Bovine | Liver | [[44](#_ENREF_44)] |
| *n3-PUFA* | PPARα | **⇑** | Bovine | Endometrium | [[45](#_ENREF_45)] |
|  |  | **⇔** |  | Embryo | [[46](#_ENREF_46)] |
|  | PPARβ/δ | **⇔** |  | Muscle | [[47](#_ENREF_47)] |
|  |  | **⇑** |  | Endometrium | [[45](#_ENREF_45)] |
|  | PPARγ | **⇔** |  | Embryo | [[46](#_ENREF_46)] |
|  |  | **⇔** |  | Endometrium | [[45](#_ENREF_45)] |
| *20:5(n-3)* | PPARγ | **⇔** | Bovine | i.m adipocytes | [[48](#_ENREF_48)] |
|  | PPARβ/δ | **⇑** |  | BEND | [[20](#_ENREF_20)] |
| *β-carotene* | PPARγ | **⇑** | Bovine | Adipocytes | [[32](#_ENREF_32)] |
| *9-cis retinoic acid* | PPARγ | **⇑** | Bovine | Adipocytes | [[32](#_ENREF_32)] |
| *All trans retinoic acid* | PPARγ | **⇑** | Bovine | Adipocytes | [[32](#_ENREF_32)] |
| *Flaxseed-feeding* | PPARγ | **⇑** | Bovine | L. dorsi muscle | [[49](#_ENREF_49)] |
|  | PPARα | **⇔** |  |  |  |
| *Melengestrol acetate* | PPARγ | **⇑** | Bovine | MDC | [[50](#_ENREF_50)] |
| *Phorbol ester* | PPARα | **⇑** | Bovine | BEND | [[20](#_ENREF_20)] |
|  | PPARβ/δ | **⇑** |  |  |  |
| *Phytanic acid* | PPARγ | **⇑** | Bovine | Adipocytes | [[32](#_ENREF_32)] |
| *Pristanic acid* | PPARγ | **⇑** | Bovine | Adipocytes | [[32](#_ENREF_32)] |
| *Physiological status* | | | | | |
| *Age* | PPARγ | **⇑** | Bovine (W)α | Adipose depots | [[51](#_ENREF_51)] |
|  |  | **⇑** | Bovine (JB)α | Muscle | [[52](#_ENREF_52)] |
|  |  | **⇑** | Bovine (A,JB,H)α |  | [[53](#_ENREF_53),[54](#_ENREF_54)] |
|  |  | **⇓⇑**^@^ | Ovine | Muscle | [[55](#_ENREF_55)] |
|  | PPARα | **⇓** |  | Left heart ventricle | [[56](#_ENREF_56)] |
| *Estrous cycle* | PPARα | **⇓** | Ovine | Endometrium | [[57](#_ENREF_57)] |
|  |  | **⇓** |  | Trophoblast |  |
|  | PPARγ | **⇓⇑^** |  | Endometrium |  |
|  |  | **⇑** |  | Trophoblast |  |
|  | PPARβ/δ | **⇔** |  | Endometrium |  |
|  |  | **⇑** |  | Trophoblast |  |
|  |  | **⇓** | Bovine | Uterine | [[58](#_ENREF_58)] |
| *Inflammation* | PPARγ | **⇓** | Bovine | Neutrophil | + |
|  |  | **⇓** |  | Mammary | [[59](#_ENREF_59)] |
|  |  | **⇔** |  | Liver | [[60](#_ENREF_60)] |
|  | PPARα | **⇔** |  | Liver | [[60](#_ENREF_60)] |
|  |  | **⇓** |  | Neutrophil | + |
|  | PPARβ/δ | **⇑** |  | Neutrophil | + |
| *Lactation* | PPARα | **⇔”** | Bovine | Mammary | [[61](#_ENREF_61)] |
|  | PPARβ/δ | **⇓** |  |  | [[61](#_ENREF_61)] |
|  | PPARγ | **⇑** |  |  | [[62](#_ENREF_62)] |
| *Peripartum* | PPARα | **⇑** | Bovine | Liver | [[63](#_ENREF_63)] |
|  |  | **⇑** |  |  | [[64](#_ENREF_64),[65](#_ENREF_65)] |
|  |  | **⇔** |  |  | [[66](#_ENREF_66)] |
|  | PPARγ | **⇔** |  |  | [[65](#_ENREF_65)] |
|  |  | **⇓** |  | Subcutan. adipose | [[67](#_ENREF_67),[68](#_ENREF_68)] |
| *Dietary energy* | | | | | |
| *Feed restriction* | PPARα | **⇑** | Ovine | Fetal adipose | [[69](#_ENREF_69)] |
|  |  | **⇑** | Bovine | Liver | [[70](#_ENREF_70)] |
|  |  | **⇑** |  | Muscle | [[71](#_ENREF_71)] |
|  | PPARβ/δ | **⇑** | Bovine | Liver | [[70](#_ENREF_70)] |
|  |  | **⇑** |  | Muscle | [[71](#_ENREF_71)] |
|  | PPARγ | **⇔** | Ovine | Fetal adipose | [[69](#_ENREF_69)] |
|  |  | **⇑** |  | Placenta^$^ | [[72](#_ENREF_72)] |
|  |  | **⇑** | Bovine | Muscle | [[71](#_ENREF_71)] |
|  |  | **⇑** |  | Hypothalamus+ | [[73](#_ENREF_73)] |
| *High dietary energy* | PPARγ | **⇑** | Bovine | Adipose | [[34](#_ENREF_34),[68](#_ENREF_68)] |
|  |  | **⇔** |  | Adipose^%^ | [[51](#_ENREF_51)] |
|  |  | **⇑** |  | Muscle | [[54](#_ENREF_54)] |
|  |  | **⇑** | Ovine | Fetal adipose | [[54](#_ENREF_54),[74](#_ENREF_74)] |
|  |  | **⇑** |  | Muscle | [[75](#_ENREF_75)] |
|  | PPARα^&^ | **⇓** | Bovine | Liver | [[76](#_ENREF_76)] |
|  | PPARβ/δ^!^ | **⇓** | Bovine | Muscle | [[77](#_ENREF_77)] |
| *Hormones/growth factors/cytokines* | | | | | |
| *Adipogenic medium^#^* | PPARγ | **⇑** | Caprine | ASC | [[78](#_ENREF_78)] |
|  |  | **⇑** | Bovine | MDC | [[50](#_ENREF_50)] |
|  |  | **⇑** |  | ASC | [[79](#_ENREF_79)] |
|  | PPARα | **⇓** | Bovine | ASC | [[79](#_ENREF_79)] |
| *Growth hormone* | PPARα | **⇑** | Bovine | BEND | [[20](#_ENREF_20)] |
|  |  | **⇓** |  | Liver | [[66](#_ENREF_66)] |
|  | PPARγ | **⇔** |  | Uterus | [[58](#_ENREF_58)] |
| *Interferon τ* | PPARβ/δ | **⇑** | Bovine | BEND | [[20](#_ENREF_20)] |
| *Lutein* | PPARγ | **⇑** | Bovine | Adipocytes | [[32](#_ENREF_32)] |
| *Synthetic PPAR agonists* | | | | | |
| *Ciglitazone* | PPARβ/δ | **⇓** | Bovine | bMEC | [[25](#_ENREF_25)] |
|  | PPARγ | **⇓** |  |  |  |
| *Fenofibrate* | PPARα | **⇔** | Bovine | Liver | [[30](#_ENREF_30)] |
| *GW0742* | PPARβ/δ | **⇑** | Bovine | BAEC | [[80](#_ENREF_80)] |
| *MC-555* | PPARβ/δ | **⇓** | Bovine | bMEC | [[25](#_ENREF_25)] |
| *PGJ2* | PPARβ/δ | **⇓** | Bovine | bMEC | [[25](#_ENREF_25)] |
| *Rosiglitazone* | PPARγ | **⇑** | Buffalo | Granulosa cells | [[43](#_ENREF_43)] |
|  |  | **⇑** | Bovine | bEPC | [[22](#_ENREF_22)] |
|  |  | **⇔** | Bovine | MAC-T | [[28](#_ENREF_28)] |
|  |  | **⇑** | Bovine | Adipocytes | [[32](#_ENREF_32)] |
|  | PPARα | **⇑** | Ovine | Liver | [[29](#_ENREF_29)] |
|  |  | **⇑** | Ovine | Muscle |  |
|  | PPARβ/δ | **⇓** | Bovine | bMEC | [[25](#_ENREF_25)] |
| *Thiazolidinedione* | PPARγ | **⇑** | Bovine | Adipose | [[35](#_ENREF_35)] |
|  |  | **⇔** |  |  | [[39](#_ENREF_39)] |
|  |  | **⇓** |  | Liver |  |
|  |  | **⇔** |  | Muscle |  |
|  | PPARα | **⇓** |  | Liver |  |
|  |  | **⇑** |  | Muscle |  |
|  | PPARβ/δ | **⇓** |  | Liver |  |
|  |  | **⇔** |  | Muscle |  |
| *Wy-14643* | PPARα | **⇔** |  | MDBK | [[24](#_ENREF_24),[27](#_ENREF_27),[38](#_ENREF_38)] |
| *Mechanical cues/others* | | | | | |
| *Low birth weight* | PPARγ | **⇑** | Ovine | Lamb visceral fat | [[81](#_ENREF_81)] |
| *Male x IUGR* | PPARγ | **⇓** | Ovine | Perirenal adipose | [[82](#_ENREF_82)] |
| *H_2_O_2_* | PPARγ | **⇓** | Bovine | bEPC, BAEC | [[22](#_ENREF_22),[83](#_ENREF_83)] |
| *Intrauterine growth restriction* | PPARγ | **⇓** | Ovine♂ | Perirenal adipose | [[82](#_ENREF_82)] |
| *Marbling capacity* | PPARγ | **⇑** | Bovine | Subcut. adipose | [[84](#_ENREF_84)] |
|  |  | **⇑** |  | Intramuscul. adipose |  |
| *Laminar flow* | PPARγ | **⇑** | Bovine | BAEC | [[85](#_ENREF_85)] |
| *Long-term hypoxia* | PPARγ | **⇑** | Ovine | Fetus adipose | [[86](#_ENREF_86)] |
|  | PPARα | **⇔** |  |  |  |
| *Mechanical load* | PPARγ | **⇓** | Bovine | ASC | [[87](#_ENREF_87)] |

^1^ Consequence of effector on mRNA expression of PPAR (**⇑** induction; **⇓** inhibition; ⇔ no change).

^2^ BAEC = Bovine Aortic Endothelial Cells; BEND = bovine endometrial cell line; bEPC = Bovine renal epithelial cells; bMEC = bovine mammary epithelial cells; CLA = conjugated linoleic acid (not isomer specified); IUGR = Intra Uterine Growth Restriction; MDBK = Madin-Darby Kidney Cell Line; MDC = bovine muscle-derived cells; MAC-T = Bovine Mammary Epithelial Cell Line; PGJ2 = prostaglandin J2.

^@^ Decreased with age in male Kazak sheep but increased in Xinjiang sheep muscle.

* Only after 18h of treatment; at 6h not change was observed in both experiments.

^+^ Unpublished data (Moyes K., Graugnard D., Khan M., Bionaz M., and Loor J.).

^$^ Only between 65 and 110 days of gestation.

^#^ For goat’s cells the differentiating medium was composed of DMEM/F12 containing 3% FBS, antibiotics, 33 μM biotin, 17 μM pantothenic acid, 1 μM insulin, 1 μM dexamethasone, 0.5 mM 3-isobutyl-1-methylxanthine (IBMX), 5 μM rosiglitazone and 5% rabbit serum for 3 days, then fed inducing medium without rosiglitazone and IBMX; for the bovine’s ASC the medium was composed by 1 g/mL insulin, 0.25 M dexamethasone, and 10 mM acetic acid in DMEM supplemented with 10% FBS; for bovine MDC the medium was composed of insulin (10 μM), oleic acid (100 μM), and ciglitizone (10 μM) in Dulbecco’s modified Eagle’s medium.

^%^ Fattening period.

^&^ The overfeeding of energy was during prepartum but decreased expression of PPARA compared to control was observed 14 days postpartum where diet was similar between groups.

^!^The lower expression of *PPARD* with high dietary energy was observed only in Angus but not in Angus×Simmental steers.

^The *PPARG* expression was decreased during the first 14 days of pregnancy but increased from 14 to 17 days of pregnancy.

+The greater expression of PPARγ was observed at the protein level but not at the transcript level.

“The temporal expression of *PPARA* in bovine mammary tissue was significant at False Discovery Rate=0.008, with the only significant observable increase at the end of lactation (e.g., 300 vs. -30d relative to parturition).

αA = Angus; W = Wagyu; JB= Japanese Black

REFERENCES

1. Lohrke B, Viergutz T, Shahi SK, Pohland R, Wollenhaupt K, et al. (1998) Detection and functional characterisation of the transcription factor peroxisome proliferator-activated receptor gamma in lutein cells. J Endocrinol 159: 429-439.

2. Ohyama M, Matsuda K, Torii S, Matsui T, Yano H, et al. (1998) The interaction between vitamin A and thiazolidinedione on bovine adipocyte differentiation in primary culture. J Anim Sci 76: 61-65.

3. Marx N, Bourcier T, Sukhova GK, Libby P, Plutzky J (1999) PPARgamma activation in human endothelial cells increases plasminogen activator inhibitor type-1 expression: PPARgamma as a potential mediator in vascular disease. Arteriosclerosis, thrombosis, and vascular biology 19: 546-551.

4. Soret B, Lee HJ, Finley E, Lee SC, Vernon RG (1999) Regulation of differentiation of sheep subcutaneous and abdominal preadipocytes in culture. J Endocrinol 161: 517-524.

5. Bishop-Bailey D, Hla T (1999) Endothelial cell apoptosis induced by the peroxisome proliferator-activated receptor (PPAR) ligand 15-deoxy-Delta12, 14-prostaglandin J2. The Journal of biological chemistry 274: 17042-17048.

6. Delerive P, Martin-Nizard F, Chinetti G, Trottein F, Fruchart JC, et al. (1999) Peroxisome proliferator-activated receptor activators inhibit thrombin-induced endothelin-1 production in human vascular endothelial cells by inhibiting the activator protein-1 signaling pathway. Circ Res 85: 394-402.

7. Hayashida K, Kume N, Minami M, Kataoka H, Morimoto M, et al. (2001) Peroxisome proliferator-activated receptor a ligands increase lectin-like oxidized low density lipoprotein receptor-1 expression in vascular endothelial cells. Ann N Y Acad Sci 947: 370-372.

8. Chiba Y, Ogita T, Ando K, Fujita T (2001) PPARgamma ligands inhibit TNF-alpha-induced LOX-1 expression in cultured endothelial cells. Biochem Biophys Res Commun 286: 541-546.

9. Fukunaga Y, Itoh H, Doi K, Tanaka T, Yamashita J, et al. (2001) Thiazolidinediones, peroxisome proliferator-activated receptor gamma agonists, regulate endothelial cell growth and secretion of vasoactive peptides. Atherosclerosis 158: 113-119.

10. Kushibiki S, Hodate K, Shingu H, Ueda Y, Shinoda M, et al. (2001) Insulin resistance induced in dairy steers by tumor necrosis factor alpha is partially reversed by 2,4-thiazolidinedione. Domest Anim Endocrinol 21: 25-37.

11. Kandoussi A, Martin F, Hazzan M, Noel C, Fruchart JC, et al. (2002) HMG-CoA reductase inhibition and PPAR- alpha activation both inhibit cyclosporin A induced endothelin-1 secretion in cultured endothelial cells. Clin Sci (Lond) 103 Suppl 48: 81S-83S.

12. Cappon GD, Liu RC, Frame SR, Hurtt ME (2002) Effects of the rat hepatic peroxisome proliferator, Wyeth 14,643, on the lactating goat. Drug Chem Toxicol 25: 255-266.

13. Froment P, Fabre S, Dupont J, Pisselet C, Chesneau D, et al. (2003) Expression and functional role of peroxisome proliferator-activated receptor-gamma in ovarian folliculogenesis in the sheep. Biol Reprod 69: 1665-1674.

14. Goya K, Sumitani S, Xu X, Kitamura T, Yamamoto H, et al. (2004) Peroxisome proliferator-activated receptor alpha agonists increase nitric oxide synthase expression in vascular endothelial cells. Arterioscler Thromb Vasc Biol 24: 658-663.

15. Hayashida K, Kume N, Minami M, Inui-Hayashida A, Mukai E, et al. (2004) Peroxisome proliferator-activated receptor alpha ligands activate transcription of lectin-like oxidized low density lipoprotein receptor-1 gene through GC box motif. Biochem Biophys Res Commun 323: 1116-1123.

16. Cho DH, Choi YJ, Jo SA, Jo I (2004) Nitric oxide production and regulation of endothelial nitric-oxide synthase phosphorylation by prolonged treatment with troglitazone: evidence for involvement of peroxisome proliferator-activated receptor (PPAR) gamma-dependent and PPARgamma-independent signaling pathways. J Biol Chem 279: 2499-2506.

17. Kim J, Oh YS, Shinn SH (2005) Troglitazone reverses the inhibition of nitric oxide production by high glucose in cultured bovine retinal pericytes. Exp Eye Res 81: 65-70.

18. Cho DH, Choi YJ, Jo SA, Ryou J, Kim JY, et al. (2006) Troglitazone acutely inhibits protein synthesis in endothelial cells via a novel mechanism involving protein phosphatase 2A-dependent p70 S6 kinase inhibition. Am J Physiol Cell Physiol 291: C317-326.

19. Wang Y, Yang Q, Yan JT, Zhao C, Cianflone K, et al. (2006) Effects of bezafibrate on the expression of endothelial nitric oxide synthase gene and its mechanisms in cultured bovine endothelial cells. Atherosclerosis 187: 265-273.

20. MacLaren LA, Guzeloglu A, Michel F, Thatcher WW (2006) Peroxisome proliferator-activated receptor (PPAR) expression in cultured bovine endometrial cells and response to omega-3 fatty acid, growth hormone and agonist stimulation in relation to series 2 prostaglandin production. Domest Anim Endocrinol 30: 155-169.

21. Sheldrick EL, Derecka K, Marshall E, Chin EC, Hodges L, et al. (2007) Peroxisome-proliferator-activated receptors and the control of levels of prostaglandin-endoperoxide synthase 2 by arachidonic acid in the bovine uterus. Biochem J 406: 175-183.

22. Sommer M, Wolf G (2007) Rosiglitazone increases PPARgamma in renal tubular epithelial cells and protects against damage by hydrogen peroxide. Am J Nephrol 27: 425-434.

23. Smith KL, Stebulis SE, Waldron MR, Overton TR (2007) Prepartum 2,4-thiazolidinedione alters metabolic dynamics and dry matter intake of dairy cows. J Dairy Sci 90: 3660-3670.

24. Bionaz M, Baumrucker CR, Shirk E, Vanden Heuvel JP, Block E, et al. (2008) Characterization of Madin-Darby bovine kidney cell line for peroxisome proliferator-activated receptors: temporal response and sensitivity to fatty acids. J Dairy Sci 91: 2808-2813.

25. Lutzow YS, Gray C, Tellam R (2008) 15-Deoxy-Delta12,14-prostaglandin J2 induces chemokine expression, oxidative stress and microfilament reorganization in bovine mammary epithelial cells. The Journal of dairy research 75: 55-63.

26. Smith KL, Butler WR, Overton TR (2009) Effects of prepartum 2,4-thiazolidinedione on metabolism and performance in transition dairy cows. J Dairy Sci 92: 3623-3633.

27. Thering BJ, Bionaz M, Loor JJ (2009) Long-chain fatty acid effects on peroxisome proliferator-activated receptor-alpha-regulated genes in Madin-Darby bovine kidney cells: optimization of culture conditions using palmitate. J Dairy Sci 92: 2027-2037.

28. Kadegowda AK, Bionaz M, Piperova LS, Erdman RA, Loor JJ (2009) Peroxisome proliferator-activated receptor-gamma activation and long-chain fatty acids alter lipogenic gene networks in bovine mammary epithelial cells to various extents. J Dairy Sci 92: 4276-4289.

29. Muhlhausler BS, Morrison JL, McMillen IC (2009) Rosiglitazone increases the expression of peroxisome proliferator-activated receptor-gamma target genes in adipose tissue, liver, and skeletal muscle in the sheep fetus in late gestation. Endocrinology 150: 4287-4294.

30. Litherland NB, Bionaz M, Wallace RL, Loor JJ, Drackley JK (2010) Effects of the peroxisome proliferator-activated receptor-alpha agonists clofibrate and fish oil on hepatic fatty acid metabolism in weaned dairy calves. J Dairy Sci 93: 2404-2418.

31. Riahi Y, Sin-Malia Y, Cohen G, Alpert E, Gruzman A, et al. (2010) The natural protective mechanism against hyperglycemia in vascular endothelial cells: roles of the lipid peroxidation product 4-hydroxydodecadienal and peroxisome proliferator-activated receptor delta. Diabetes 59: 808-818.

32. Garcia-Rojas P, Antaramian A, Gonzalez-Davalos L, Villarroya F, Shimada A, et al. (2010) Induction of peroxisomal proliferator-activated receptor gamma and peroxisomal proliferator-activated receptor gamma coactivator 1 by unsaturated fatty acids, retinoic acid, and carotenoids in preadipocytes obtained from bovine white adipose tissue1,2. J Anim Sci 88: 1801-1808.

33. Lengi AJ, Corl BA (2010) Factors influencing the differentiation of bovine preadipocytes in vitro. J Anim Sci 88: 1999-2008.

34. Schoenberg KM, Overton TR (2011) Effects of plane of nutrition and 2,4-thiazolidinedione on insulin responses and adipose tissue gene expression in dairy cattle during late gestation. J Dairy Sci 94: 6021-6035.

35. Schoenberg KM, Perfield KL, Farney JK, Bradford BJ, Boisclair YR, et al. (2011) Effects of prepartum 2,4-thiazolidinedione on insulin sensitivity, plasma concentrations of tumor necrosis factor-alpha and leptin, and adipose tissue gene expression. J Dairy Sci 94: 5523-5532.

36. Werner C, Gensch C, Poss J, Haendeler J, Bohm M, et al. (2011) Pioglitazone activates aortic telomerase and prevents stress-induced endothelial apoptosis. Atherosclerosis 216: 23-34.

37. Perdomo MC, Santos JE, Badinga L (2011) Trans-10, cis-12 conjugated linoleic acid and the PPAR-gamma agonist rosiglitazone attenuate lipopolysaccharide-induced TNF-alpha production by bovine immune cells. Domest Anim Endocrinol 41: 118-125.

38. Bionaz M, Thering BJ, Loor JJ (2012) Fine metabolic regulation in ruminants via nutrient-gene interactions: saturated long-chain fatty acids increase expression of genes involved in lipid metabolism and immune response partly through PPAR-alpha activation. The British journal of nutrition 107: 179-191.

39. Arevalo-Turrubiarte M, Gonzalez-Davalos L, Yabuta A, Garza JD, Davalos JL, et al. (2012) Effect of 2,4-thiazolidinedione on limousin cattle growth and on muscle and adipose tissue metabolism. PPAR Res 2012: 891841.

40. Sharma S, Sun X, Rafikov R, Kumar S, Hou Y, et al. (2012) PPAR-gamma regulates carnitine homeostasis and mitochondrial function in a lamb model of increased pulmonary blood flow. PLoS One 7: e41555.

41. Lee SH, Hossner KL (2002) Coordinate regulation of ovine adipose tissue gene expression by propionate. J Anim Sci 80: 2840-2849.

42. Selberg KT, Staples CR, Luchini ND, Badinga L (2005) Dietary trans octadecenoic acids upregulate the liver gene encoding peroxisome proliferator-activated receptor-alpha in transition dairy cows. J Dairy Res 72: 107-114.

43. Sharma I, Monga R, Singh N, Datta TK, Singh D (2012) Ovary-specific novel peroxisome proliferator activated receptors-gamma transcripts in buffalo. Gene 504: 245-252.

44. Sigl T, Schlamberger G, Kienberger H, Wiedemann S, Meyer HH, et al. (2010) Rumen-protected conjugated linoleic acid supplementation to dairy cows in late pregnancy and early lactation: effects on milk composition, milk yield, blood metabolites and gene expression in liver. Acta Vet Scand 52: 16.

45. Coyne GS, Kenny DA, Childs S, Sreenan JM, Waters SM (2008) Dietary n-3 polyunsaturated fatty acids alter the expression of genes involved in prostaglandin biosynthesis in the bovine uterus. Theriogenology 70: 772-782.

46. Childs S, Carter F, Lynch CO, Sreenan JM, Lonergan P, et al. (2008) Embryo yield and quality following dietary supplementation of beef heifers with n-3 polyunsaturated fatty acids (PUFA). Theriogenology 70: 992-1003.

47. Waters SM, Kelly JP, O'Boyle P, Moloney AP, Kenny DA (2009) Effect of level and duration of dietary n-3 polyunsaturated fatty acid supplementation on the transcriptional regulation of Delta9-desaturase in muscle of beef cattle. J Anim Sci 87: 244-252.

48. Waters SM, Kenny DA, Killeen AP, Spellman SA, Fitzgerald A, et al. (2009) Effect of level of eicosapentaenoic acid on the transcriptional regulation of Delta-9 desaturase using a novel in vitro bovine intramuscular adipocyte cell culture model. Animal 3: 718-727.

49. Kronberg SL, Barcelo-Coblijn G, Shin J, Lee K, Murphy EJ (2006) Bovine muscle n-3 fatty acid content is increased with flaxseed feeding. Lipids 41: 1059-1068.

50. Chung KY, Johnson BJ (2009) Melengestrol acetate enhances adipogenic gene expression in cultured muscle-derived cells. J Anim Sci 87: 3897-3904.

51. Yamada T, Kawakami SI, Nakanishi N (2007) Effects of fattening periods on the expression of adipogenic transcription factors in Wagyu beef cattle. Meat science 76: 289-294.

52. Shibata M, Matsumoto K, Hikino Y, Oe M, Ojima K, et al. (2011) Influence of different feeding systems on the growth performance and muscle development of Japanese Black steers. Meat Sci 89: 451-456.

53. Albrecht E, Gotoh T, Ebara F, Xu JX, Viergutz T, et al. (2011) Cellular conditions for intramuscular fat deposition in Japanese Black and Holstein steers. Meat Sci 89: 13-20.

54. Graugnard DE, Berger LL, Faulkner DB, Loor JJ (2010) High-starch diets induce precocious adipogenic gene network up-regulation in longissimus lumborum of early-weaned Angus cattle. Br J Nutr 103: 953-963.

55. Huang ZG, Xiong L, Liu ZS, Qiao Y, Liu SR, et al. (2006) The developmental changes and effect on IMF content of H-FABP and PPARgamma mRNA expression in sheep muscle. Yi Chuan Xue Bao 33: 507-514.

56. McClure TD, Young ME, Taegtmeyer H, Ning XH, Buroker NE, et al. (2005) Thyroid hormone interacts with PPARalpha and PGC-1 during mitochondrial maturation in sheep heart. Am J Physiol Heart Circ Physiol 289: H2258-2264.

57. Cammas L, Reinaud P, Bordas N, Dubois O, Germain G, et al. (2006) Developmental regulation of prostacyclin synthase and prostacyclin receptors in the ovine uterus and conceptus during the peri-implantation period. Reproduction 131: 917-927.

58. Balaguer SA, Pershing RA, Rodriguez-Sallaberry C, Thatcher WW, Badinga L (2005) Effects of bovine somatotropin on uterine genes related to the prostaglandin cascade in lactating dairy cows. J Dairy Sci 88: 543-552.

59. Mitterhuemer S, Petzl W, Krebs S, Mehne D, Klanner A, et al. (2010) Escherichia coli infection induces distinct local and systemic transcriptome responses in the mammary gland. BMC Genomics 11: 138.

60. Jiang L, Sorensen P, Rontved C, Vels L, Ingvartsen KL (2008) Gene expression profiling of liver from dairy cows treated intra-mammary with lipopolysaccharide. BMC Genomics 9: 443.

61. Bionaz M, Periasamy K, Rodriguez-Zas SL, Hurley WL, Loor JJ (2012) A Novel Dynamic Impact Approach (DIA) for Functional Analysis of Time-Course Omics Studies: Validation Using the Bovine Mammary Transcriptome. PloS one 7: e32455.

62. Bionaz M, Loor JJ (2008) Gene networks driving bovine milk fat synthesis during the lactation cycle. BMC Genomics 9: 366.

63. Loor JJ, Dann HM, Everts RE, Oliveira R, Green CA, et al. (2005) Temporal gene expression profiling of liver from periparturient dairy cows reveals complex adaptive mechanisms in hepatic function. Physiol Genomics 23: 217-226.

64. Schlegel G, Keller J, Hirche F, Geissler S, Schwarz FJ, et al. (2012) Expression of genes involved in hepatic carnitine synthesis and uptake in dairy cows in the transition period and at different stages of lactation. BMC Vet Res 8: 28.

65. van Dorland HA, Richter S, Morel I, Doherr MG, Castro N, et al. (2009) Variation in hepatic regulation of metabolism during the dry period and in early lactation in dairy cows. J Dairy Sci 92: 1924-1940.

66. Carriquiry M, Weber WJ, Fahrenkrug SC, Crooker BA (2009) Hepatic gene expression in multiparous Holstein cows treated with bovine somatotropin and fed n-3 fatty acids in early lactation. J Dairy Sci 92: 4889-4900.

67. Schmitt E, Ballou MA, Correa MN, DePeters EJ, Drackley JK, et al. (2011) Dietary lipid during the transition period to manipulate subcutaneous adipose tissue peroxisome proliferator-activated receptor-gamma co-regulator and target gene expression. Journal of dairy science 94: 5913-5925.

68. Ji P, Osorio JS, Drackley JK, Loor JJ (2012) Overfeeding a moderate energy diet prepartum does not impair bovine subcutaneous adipose tissue insulin signal transduction and induces marked changes in peripartal gene network expression. Journal of Dairy Science 95: 4333-4351.

69. Bispham J, Gardner DS, Gnanalingham MG, Stephenson T, Symonds ME, et al. (2005) Maternal nutritional programming of fetal adipose tissue development: differential effects on messenger ribonucleic acid abundance for uncoupling proteins and peroxisome proliferator-activated and prolactin receptors. Endocrinology 146: 3943-3949.

70. Loor JJ, Everts RE, Bionaz M, Dann HM, Morin DE, et al. (2007) Nutrition-induced ketosis alters metabolic and signaling gene networks in liver of periparturient dairy cows. Physiol Genomics 32: 105-116.

71. Brennan KM, Michal JJ, Ramsey JJ, Johnson KA (2009) Body weight loss in beef cows: I. The effect of increased beta-oxidation on messenger ribonucleic acid levels of uncoupling proteins two and three and peroxisome proliferator-activated receptor in skeletal muscle. J Anim Sci 87: 2860-2866.

72. Yiallourides M, Sebert SP, Wilson V, Sharkey D, Rhind SM, et al. (2009) The differential effects of the timing of maternal nutrient restriction in the ovine placenta on glucocorticoid sensitivity, uncoupling protein 2, peroxisome proliferator-activated receptor-gamma and cell proliferation. Reproduction 138: 601-608.

73. Kuhla B, Gors S, Metges CC (2011) Hypothalamic orexin A expression and the involvement of AMPK and PPAR-gamma signalling in energy restricted dairy cows. Archiv Fur Tierzucht-Archives of Animal Breeding 54: 567-579.

74. Muhlhausler BS, Duffield JA, McMillen IC (2007) Increased maternal nutrition stimulates peroxisome proliferator activated receptor-gamma, adiponectin, and leptin messenger ribonucleic acid expression in adipose tissue before birth. Endocrinology 148: 878-885.

75. Tong J, Zhu MJ, Underwood KR, Hess BW, Ford SP, et al. (2008) AMP-activated protein kinase and adipogenesis in sheep fetal skeletal muscle and 3T3-L1 cells. J Anim Sci 86: 1296-1305.

76. Janovick-Guretzky NA, Dann HM, Loor JJ, Drackley JK (2007) Prepartum plane of dietary energy alters hepatic expression of inflammatory and fatty acid oxidation genes in dairy cows. Faseb Journal 21: A374-A374.

77. Graugnard DE, Piantoni P, Bionaz M, Berger LL, Faulkner DB, et al. (2009) Adipogenic and energy metabolism gene networks in longissimus lumborum during rapid post-weaning growth in Angus and Angus x Simmental cattle fed high-starch or low-starch diets. BMC Genomics 10: 142.

78. Ren Y, Wu H, Zhou X, Wen J, Jin M, et al. (2012) Isolation, expansion, and differentiation of goat adipose-derived stem cells. Research in veterinary science 93: 404-411.

79. Taniguchi M, Guan LL, Zhang B, Dodson MV, Okine E, et al. (2008) Gene expression patterns of bovine perimuscular preadipocytes during adipogenesis. Biochemical and biophysical research communications 366: 346-351.

80. Fan Y, Wang Y, Tang Z, Zhang H, Qin X, et al. (2008) Suppression of pro-inflammatory adhesion molecules by PPAR-delta in human vascular endothelial cells. Arteriosclerosis, thrombosis, and vascular biology 28: 315-321.

81. Muhlhausler BS, Ritorto V, Schultz C, Chatterton BE, Duffield JA, et al. (2008) Birth weight and gender determine expression of adipogenic, lipogenic and adipokine genes in perirenal adipose tissue in the young adult sheep. Domest Anim Endocrinol 35: 46-57.

82. Duffield JA, Vuocolo T, Tellam R, McFarlane JR, Kauter KG, et al. (2009) Intrauterine growth restriction and the sex specific programming of leptin and peroxisome proliferator-activated receptor gamma (PPARgamma) mRNA expression in visceral fat in the lamb. Pediatr Res 66: 59-65.

83. Blanquicett C, Kang BY, Ritzenthaler JD, Jones DP, Hart CM (2010) Oxidative stress modulates PPAR gamma in vascular endothelial cells. Free Radic Biol Med 48: 1618-1625.

84. Bonnet M, Faulconnier Y, Leroux C, Jurie C, Cassar-Malek I, et al. (2007) Glucose-6-phosphate dehydrogenase and leptin are related to marbling differences among Limousin and Angus or Japanese Black x Angus steers. J Anim Sci 85: 2882-2894.

85. Liu Y, Zhu Y, Rannou F, Lee TS, Formentin K, et al. (2004) Laminar flow activates peroxisome proliferator-activated receptor-gamma in vascular endothelial cells. Circulation 110: 1128-1133.

86. Myers DA, Hanson K, Mlynarczyk M, Kaushal KM, Ducsay CA (2008) Long-term hypoxia modulates expression of key genes regulating adipose function in the late-gestation ovine fetus. Am J Physiol Regul Integr Comp Physiol 294: R1312-1318.

87. David V, Martin A, Lafage-Proust MH, Malaval L, Peyroche S, et al. (2007) Mechanical loading down-regulates peroxisome proliferator-activated receptor gamma in bone marrow stromal cells and favors osteoblastogenesis at the expense of adipogenesis. Endocrinology 148: 2553-2562.
